# Modern plant stress adaptation: integrating defense, nanotechnology and genetics

**DOI:** 10.1007/s13205-026-04732-z

**Published:** 2026-02-20

**Authors:** Meenakshi, Komal, Arun Sharma, Sudharshan Prabhu, Shikha Awasthi

**Affiliations:** 1 Department of Botany, Career Point University, Kota, Rajasthan India; 2 Department of Chemistry, School of Physical and Biosciences, Manipal University Jaipur, Jaipur, 303007 Rajasthan India; 3 Department of Chemistry, Career Point University, Kota, Rajasthan India; 4 Department of Cellular and Molecular Biology, Manipal School of Life Sciences, Manipal Academy of Higher Education, 576104 Manipal, Karnataka India; 5 Jagran School of Pharmacy, Jagran Lakecity University, Bhopal, 462044 Madhya Pradesh India

**Keywords:** CRISPR, Plant immunity, ROS signaling, Genetic mechanisms & antioxidant modulation

## Abstract

This review critically analyses plant adaptive responses to biotic and abiotic stress, with a focus on recent advancements in molecular defense pathways, emerging nanotechnology approaches and CRISPR/Cas-based genome editing strategies. We critically reviewed structural, physiological, biochemical and genetic adaptations. Key regulatory processes include phytohormonal regulation, antioxidants, reactive oxygen species (ROS) signaling and stress-response gene networks are explored along with advances in nanotechnology-based strategies and CRISPR/Cas genome editing. A comparative evaluation of conventional breeding, molecular breeding, and genome-editing approaches highlights the advantages of CRISPR/Cas systems, particularly their precision, efficiency and ability to generate targeted phenotypic changes. In parallel, nanomaterials have shown promise in improved nutrient delivery, protecting cellular structures and enhancing genome-editing efficiency under stress conditions. By integrating nanotechnology and genome-editing approaches with traditional agricultural practices, it may be possible to enhance plant resilience, sustain crop productivity and reduce reliance on chemical inputs. Overall, this review provides a cohesive perspective on how these technologies can be combined to support future crop improvement efforts to tackle climate-induced agricultural challenges.

## Introduction

Plant stress refers to a condition in which external factors interfere with normal plant growth, development, and productivity, which often arises from rapid and unfavorable environmental changes (Katsoulas et al. 2016). Broadly, plant stress is classified into two types: abiotic and biotic. Abiotic stress originates from non-living factors such as drought, salinity, temperature extremes, and nutrient imbalance, whereas biotic stress is caused by living organisms, including pathogens, pests, and herbivores (Gull et al. 2019). To survive under adverse stress conditions, plants have evolved highly coordinated adaptive responses at physiological, biochemical, and molecular levels. These responses involve dynamic changes in gene expression, modulation of hormone and reactive oxygen species (ROS) signaling, activation of immune defense pathways, and production of secondary metabolites (Shiade et al. 2024). While such mechanisms help plants to acclimatize to stress, the severity and duration of exposure largely determine whether plants recover successfully or suffer irreversible yield losses (Adhikari et al. 2022). Among the various challenges biotic stresses pose particularly complex threats. Pathogens such as fungi, bacteria, and viruses deploy diverse strategies, including toxin production, secretion of effector molecules, or suppress the host immune system to overcome plant defense mechanisms. These interactions often lead to disease development, resulting in reduced plant vigor and significantly lower yield (Chaudhary et al. 2022).

Herbivory directly suppresses photosynthetic capacity and regulated defense response, while parasitic weeds *Striga* and *Cuscuta* extract deplete host metabolic resources, significantly impacting crop productivity (Rahman Shah et al. 2023). Under stress, weeds and non-obligate microbes can also compete or change, which makes crops even less resilient (Liu et al. 2025). Because chemical control has limitations, sustainable methods like biocontrol microbes, molecular breeding, nanotechnology, and CRISPR (Clustered regularly interspaced short palindromic repeats) are increasingly adopted. All of these methods increase stress tolerance in crops. These approaches collectively enhance stress tolerance in crops and keep a steady supply of food. Biocontrol agents sustainably supress pathogen sustainably, molecular breeding produces tolerant varieties, nanotechnology enables smart delivery of agrochemicals and defense molecules, and CRISPR-based editing allows precise modification of plant stress-response genes. Chemical pesticides can provide short-term disease control, but they pose risk for environment, human health and can accelerate resistance. So, it is important to use sustainable approaches. These methods work well together to improve crop stress tolerance, less reliant on chemicals, and more likely to have food security in the long term through sustainable agriculture (Tyagi et al. 2024). Nanoparticles are used to improve plant growth, biomass, water and nutrient adsorption, photosynthesis, and antioxidant defense, while mitigating oxidative damage and lipid peroxidation. They can also promote osmolyte accumulation and secondary metabolite production, influence phytohormone biosynthesis, which makes plants more resistant to drought. Drought stress greatly reduces plant growth and yield. However, carbon-based nanomaterials (MWNTs, SWNTs, graphene, fullerene) and metal-based nanoparticles (Ag, Au, Cu, Fe₂O₃, TiO₂, ZnO) may be able to help lessen its effect (Chandrashekar et al. 2023). Wheat (*Triticum aestivum*) is an important staple food crop that feeds a large proportion of global population around the world and keeps them safe from hunger. Biotic stresses like pests, pathogens, and weeds, as well as abiotic stresses like drought, salinity, and heat, can hurt its productivity. All of these factors together cause major economic losses and substantial yield reductions worldwide. Nanotechnology provides promising solutions via nanofertilizers, nanopesticides, nanoherbicides, nanosensors, and intelligent delivery systems for regulated agrochemical release. Although nanoproducts show promise in improving plant growth, stress tolerance, and grain quality, their biological effects are closely linked to their uptake, translocation and fate within plants, raising important concerns regarding potential toxicity (Kashyap et al. 2020).

*Alternaria alternata*, *Puccinia graminis*, and *Fusarium oxysporum* are important plant pathogenic fungi known for their ecological diversity and significant impact on crop health. Most fungal plant pathogens belong to the phyla Ascomycetes and Basidiomycetes and employ distinct infection strategies to colonize host plants. These pathogens typically follow either a necrotrophic or biotrophic mode of infection. Biotrophic fungi invade living host cells, suppresses defense responses and redirect host metabolism to support growth. In contrast, necrotrophic fungi actively kill host tissues and derive nutrients from dead cells. To overcome plant defense response, these pathogens form specialized infection structures and secrete virulence factors, such as toxins in necrotrophs and effector proteins in biotrophs (Doehlemann et al. 2017). Bacterial pathogens like *Pseudomonas syringae* cause major crop disease, thereby improving yields and making farming more productive all over the world (Rooney et al. 2019). These pathogens interfere with plant physiology by infecting plant tissue (apoplast) and releasing virulence factors that suppresses host immunity (Asif et al. 2024). Quorum sensing, effector proteins, and immune suppression are among the strategies used to infect hosts. Viral infections have a major impact on plant health, leading to a wide range of symptoms that impair growth, development, and overall productivity (Khan et al. 2024). These infections lead to lower yield and reduced crop quality, which have serious economic effects on farming and make it harder to feed the world’s people (Afzal and Mukhtar 2024). Phytochromes are light-sensitive receptors that control how plants grow and adapt by regulating transcription factors activity and post-transcriptional processes in *Arabiodopsis* such as promoter selection, RNA splicing, and translation (Legris et al. 2019). It also controls plant cultivation in different conditions at different stages of their lives. Light-induced conformational alteration enables phytochromes to reprogram transcription and regulate promoter activity, RNA splicing, and translation (Legris et al. 2019).

### CRISPR-based stress adaptation

CRISPR/Cas-mediated genome editing has transformed biological research and plant breeding, yet it remains nascent in extensive chromosome restructuring. It allows for duplications, inversions, and translocations, which change the order of genes and their connections (Abdul Aziz and Masmoudi 2025). These rearrangements are primarily mediated through nonhomologous end joining (NHEJ) pathways in somatic cells, which differ from natural recombination processes occurring during meiosis. Classical NHEJ (cNHEJ) stop rearrangement, while alternative NHEJ (aNHEJ) promotes them. cNHEJ is necessary for tandem duplications but aNHEJ helps with translocations, inversions, and patch insertions. This technology has shown great promises for changing the way in future crop breeding, as demonstrated in *Arabidopsis* and maize (Gehrke et al. 2021). CRISPR-Cas9 is a cutting-edge genome-editing tool that induce precise changes in plant DNA. This technique is based on a natural defense mechanism in bacteria, which protects bacteria from viruses. CRISPR sequences act as a genetic memory of invading viruses, and the Cas9 protein works as molecular scissors (Saeed et al. 2025). In genetic engineering, a guide RNA is synthesized to fit a certain DNA sequence, which guides Cas9 to a defined locus in the genome. After Cas9 cuts the DNA, the cell’s natural repair pathway either turns off the gene that was eliminated or allows insertion of new genetic material. This certain technology has changed plant science by enhancing yield, stress tolerance, disease resistance, nutritional quality. This helps to promote sustainable agriculture and food security (Bhuvaneswari et al. 2020).

RNA interference (RNAi) and CRISPR-Cas9, are advanced biotechnology tools that can help plant fight off herbivores. They are promising solutions for sustainable agriculture (Halder et al. 2022). ROS are inevitable by-products of aerobic metabolism, but they also play a central role in regulating plant immune response. Depending on their site of production and concentration, ROS can either protect plant cells or cause cellular damage. Major ROS species include superoxide (O₂⁻), singlet oxygen (¹O₂), hydroxyl radicals (·OH), and hydrogen peroxide (H₂O₂). These molecules can directly inhibit pathogens and also function as signaling molecules (Sikder et al. 2025). A rapid oxidative burst of ROS initiates the hypersensitive response (HR), a programmed cell death mechanism that restricts pathogen spread at the infection site. Beyond local defense, ROS also play a role in systemic acquired resistance (SAR) by activating long-distance signaling networks that enhance immune preparedness in uninfected tissues. Moreover, ROS interact closely with phytohormones like salicylic acid (SA), jasmonic acid (JA), and ethylene (ET) to generate both local and systemic defenses responses. Understanding these interconnected signaling pathways is essential for improving crop resilience to biotic stress (Haghpanah et al. 2025). Under both biotic and abiotic stress conditions, plants generate ROS in distinct cellular compartments, where they act as important secondary messengers regulating cellular functions. Recent studies have demonstrated that ROS production in the apoplast is tightly controlled during microbe-triggered immunity and is also modulated by abscisic acid (ABA), highlighting the precise control of ROS signaling during stress responses (Qi et al. 2017).

Climate change, population growth and increasing food waste pose a major challenge to global agriculture. They affect crop yield, soil health, and biodiversity. In this context, biostimulants have emerged as a sustainable and environmentally friendly strategy to increase crop yield while reducing dependency on chemical fertilizers. Biostimulants, including plant growth-promoting microorganisms and microalgae-derived extracts, improve nutrient-use efficiency, stimulate plant growth and enhance tolerance to both biotic and abiotic stresses. By supporting plant resistance and optimizing nutrient uptake, these natural products contribute to sustained agricultural practices and long-term food security (Brito-Lopez et al. 2025).

Plant stress responses to biotic challenges often display visible symptoms such as wilting, tissue necrosis, leaf discoloration, stunted growth, and structural deformities (Jiang and Zhou 2023). Among biotic stress factors, herbivory represents a major threat to agriculture productivity as the consumption of plant tissues by insects and animals directly reduces biomass and growth. Herbivores may attack different parts of plants including leaves, stems, roots, and reproductive structures leading to substantial physiological and developmental damage. For example, leaf-chewing insects such as caterpillars impair photosynthetic capacity by damaging leaf tissues, ultimately slowing plant growth and reducing yield potential.

Nematodes or insects that survive in the soil digest plant roots, which makes it harder for plants to take in nutrients and water. Crop plants are particularly susceptible as domestication has occasionally (Fernandez et al. 2021). In natural ecosystems, herbivory plays an important role to maintain biodiversity and regulate plant populations, but in farming, it costs a lot of money. To reduce herbivory, researchers have come up with integrated pest management strategies that include biological control, genetic engineering for resistance, and sustainable farming methods (Romeis et al. 2019). But these methods are not always effective and they depend on environment that herbivores adapt and this shows more research is needed in this area (Pringle et al. 2023). *Cuscuta* (dodder) and *Striga* (witchweed) parasitic plants are major agricultural pests. Parasitic plants attach themselves to host plants to extract water and nutrients, leading to substantial reduction in crop productivity. In severe infestations, these parasitic interactions can suppress plant growth, impair photosynthesis activity and ultimately result in plant death. *Striga* species are particularly destructive to cereal crops such as maize, sorghum, and millet, where soil fertility is low, resulting in significant yield losses (David et al. 2022). Similarly *Cuscuta* species parasitizes a wide variety of host plants, displaying normal physiological processes and significantly reducing crop yields (Stanley et al. 2021).

### Synergistic role of nanotechnology and CRISPR-based nanogenomics in plant improvement

The green revolution of the 1960s played a pivotal role in stabilizing global food supplies through introduction of high-yielding crop varieties, increasing use of fertilizers, and strengthened agricultural institutions. These advances significantly reduced malnutrition and poverty in many regions of world (Bailey-Serres et al. 2019). Building on this foundation, modern crop improvement strategies such as CRISPR/Cas9 genome-editing system offer powerful tools to address yield losses driven by climate change, water scarcity, biotic and abiotic stresses, and soil degradation (Ndudzo et al. 2024). Crops including grains, fruits, vegetables, and ornamentals are essential for human nutrition and their sustainable improvement is critical for future food security. CRISPR technology enables precise genome modification without introducing foreign genetic material, allowing targeted trait enhancement. In parallel nanotechnology contributes for the development of nano-fertilizers, pesticides, and growth-promoting formulations that improve resource-use efficiency. When combined, genome editing and nanotechnology provide complementary strategies to enhance crop productivity and resistance, offering promising solution to global food shortages (Naik et al. 2022).

Despite considerable potential of CRISPR/Cas system, the widespread use in plant biotechnology still faces challenges related to delivery efficiency, species specificity and transformation successes. Nanotechnology offers potential solutions to these limitations by facilitating CRISPR cargo delivery, enabling species-independent gene transfer, enhancing germline transformation, and improving overall editing efficiency. Continued development of nanomaterial-based delivery platforms will be essential to accelerate the adoption of genome editing in crops, thereby supporting global food production and strengthening agricultural resilience to climate change (Demirer et al. 2021).

Nanomaterials are used to enhance CRISPR delivery into wide range of plant species such as *Arabidopsis*, tobacco, maize, wheat, spinach, cotton, and watercress. These nanomaterials can enter plant cells through physical or mechanical means, enabling genome-editing cargo delivery largely independent of the plant’s genetic background (Demirer et al. 2021). CRISPR-Cas genome editing tool is used to develop crops that can handle stress. It is faster and more accurate than traditional breeding. Some examples are CRISPR-Cpf1, base editing, prime editing, epigenome editing, tissue-specific (CRISPR-TSKO), and inducible genome editing (Chen et al. 2024). These tools help make cultivars that are better able to handle abiotic stresses like drought, heat and salinity. This helps food security and sustainable agriculture. Innovations like editing without tissue culture or DNA make CRISPR technologies in plant breeding (Chennakesavulu et al. 2022). Targeted genome editing in plants utilising Cas9-RNPs has demonstrated moderate efficacy previously. This study shows that using Cas9-RNP tests on wheat protoplasts makes it easy to quickly choose the best guide RNAs. It also shows that treating protoplasts and immature embryos at high temperatures speeds up the editing process even more. Edited wheat plants can be regenerated quickly and easily without adding DNA from outside sources. This creates knockouts in several homoeologous genes and pathogenic effector susceptibility genes, making the plants resistant to disease. These advances create a very effective way to edit genes without using DNA, which could speed up the process of adding new traits to crops (Poddar et al. 2023).

DNA origami (folded DNA nanostructures) is a nanocarrier that carries the CRISPR/Cas9 complex into living cells. This origami has a PAM-rich surface that can catch Cas9/sgRNA complexes and DNA/RNA hybridisation. Disulfide-linked locking strands stabilize the loaded nanostructure even more, and a DNA aptamer and an influenza HA peptide make it possible for targeted delivery and escape from the endosome. When glutathione (GSH) reduces the DNA origami inside the cell, it opens up and releases the sgRNA/Cas9 complex through RNase H-mediated cleavage. This approach enables effective editing of genes associated with tumor development, highlighting DNA origami as a rationally designed and versatile nanocarrier for CRISPR-based gene therapy (Tang et al. 2023). For plant genetic transformation and crop protection, molecular tools such as miRNA, RNAi, and CRISPR/Cas systems are widely employed. However, conventional delivery methods often have limitations including transformation efficiency, tissue damage and degradation of biomolecules.

Nanotechnology offers effective alternatives by employing a range of nanoparticle platforms including polymeric, metallic, magnetic, silica-based and carbon-based nanomaterials to improve delivery efficiency while minimizing cellular damage (Mathur et al. 2025; Mujtaba et al. 2021). Effective plant genetic transformation depends on both efficient cargo delivery and successful regeneration of edited tissues. Regeneration remains a major bottleneck due to challenges such as tissue browning, necrosis, recalcitrance, and the need for different protocols for different species (Dan 2008).

### **Impact of parasitic plants and epiphytes**

**Fig. 1:**
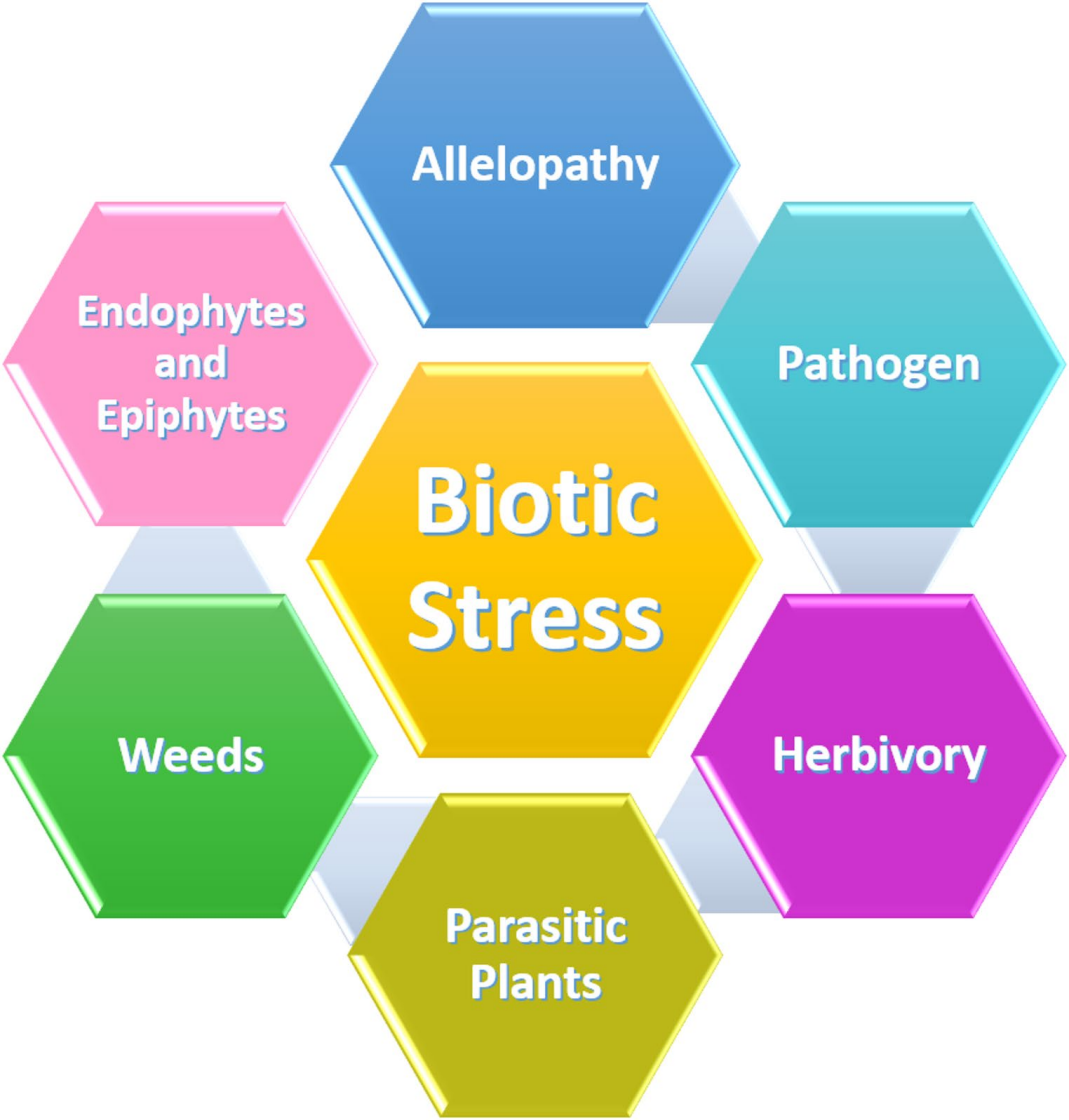
Schematic overview of the major sources of biotic stress affecting plant growth, physiology, and productivity. Biotic stress originates from living organisms, including pathogens (bacteria, fungi, viruses, and nematodes), herbivores (insects and vertebrates), parasitic plants that extract water and nutrients from host tissues, and weeds competing for space, nutrients, light, and water. Additional contributors include endophytes and epiphytes that modulate plant physiological and signaling pathways, as well as allelopathy, in which plants release allelochemicals that suppress the growth of neighbouring species, highlighting the multifactorial nature of biotic stress and its cumulative impact on plant health and crop productivity

To fight these parasitic weeds effectively, integrated strategies such as use of resistant crop varieties and appropriate cultural practices are required. However, their widespread distribution and ability to adapt to diverse situations often limit the long-term effectiveness of these approaches. Molecular breeding and biocontrol are two promising approaches to mitigate the effects of parasitic plants on crop production. Weeds intensify plant stress by competing with crops for essential resources like light, water, and nutrients, ultimately lowering productivity (Monteiro and Santos 2022). Endophytes and epiphytes generally exist in beneficial association with plants. However, under stress conditions, these relationships can shift towards harmful interactions. Endophytes can become harmful while excessive epiphytic growth can block sunlight or increase the load on host plant, negatively affecting growth and development (Fadiji and Babalola 2020). A better understanding of these diverse stress factors is essential for improving plant resilience and developing effective crop management strategies (Fig. 1).

### Sustainable disease management strategies in plants

Biocontrol is a sustainable alternative to chemical methods. It relies on microbial antagonists to suppress plant pathogens through mechanisms like hyperparasitism, predation, antibiosis, competition, and induced host resistance. Despite its potential, the effectiveness of biocontrol can be inconsistent due to environmental changes that influence survival and activity of microorganisms. This limitation has driven research into more stable formulations and integration of new technologies from biotechnology and nanotechnology for effective and reliable performance of biocontrol agents. With growing interest in sustainable farming, biocontrol approaches show significant potential for managing plant diseases, improving crop yields, and conserving natural ecosystems (Haq et al. 2024).

Plants themselves have evolved a diverse array of defense strategies at morphological, anatomical, molecular, biochemical, and genetic levels to address these challenges. However, the widespread use of copper-based bactericides and antibiotics has raised serious concern due to declining effectiveness and their adverse impact on environmental sustainability and human health. Hence, sustainable alternatives such as abiotic elicitors are gaining attention for management of bacterial diseases. These non-biological compounds enhance plant immunity by stimulating the production of phytoalexins and pathogenesis-related proteins, while also activating systemic acquired resistance (SAR) and induced systemic resistance (ISR) pathways (Zehra et al. 2021). However, to fully harness their potential for crop protection and food security, challenges related to application, optimization, mechanistic understanding and integration into existing agricultural practices must be addressed.

Effective control of plant viral diseases, similarly, requires an integrated approach that combines cultural practices to limit virus spread, develop virus-resistant crop varieties and design antiviral agents targeting specific viral groups (Akhtar et al. 2024). Advancing our understanding of plant-virus interaction and applying innovative control strategies will be critical for mitigating the impact of viral diseases and strengthening global food security (Bi et al. 2024).

## Natural plant defense mechanism

Plants have evolved advanced defense systems that can detect and respond to both biotic and abiotic stressors, allowing them to survive under a wide range of environmental threats. These defense mechanisms are broadly classified into constitutive (pre-formed) and induced responses and encompass structural barriers, chemical defense, molecular signaling networks and stress responsive pathways (Shukla et al. 2024). Together, these components function in a coordinated pattern, highlighting the interplay between physical barriers, chemical deterrents, and molecular signaling. A deeper understanding of these strategies is critical for sustainable farming because it makes it possible to create resistant crop varieties and eco-friendly ways to control pests (Zhou et al. 2024).

### Structural and chemical defenses

Plants possess a wide range of inherent structural defenses that protect them from both insect herbivores and microbial pathogens. These include lignified cell walls, cuticular waxes, thorns, and trichomes, which play a crucial role in preventing pathogens entry and limiting damage from herbivores. Thickened cuticles, lignified cell walls, and trichomes act as a primary barrier that reduce water loss and protect from environmental stress (Jeffree 2018).

In addition to physical defenses, plant synthesize diverse array of secondary metabolites such as alkaloids, phenolics, and terpenoids, which deter or eliminate herbivores and pathogens away or kill them (Zhou et al. 2024). Beyond these classic defenses, plants also influence their surrounding through chemical interactions. For example, allelopathy involves the release of bioactive compounds released into the rhizosphere that inhibit the growth of neighboring plants or soil microorganisms (Afzal et al. 2025). Plants also rely on biochemical mechanisms that help cellular stablity under stress, including accumulation of osmoprotectants, antioxidants, and other secondary metabolites that protect cells from oxidative damage and preserve metabolic balance (Anjali et al. 2023). In contrast to these self-defense strategies, parasitic plants develop specialized haustorial structures that penetrate host tissues to extract water, minerals, and photosynthates, often resulting in substantial reductions in host vigor and crop productivity (Runyon et al. 2010).

### Molecular and immune responses

Plants sense biotic stress through a complex network of receptor proteins that recognize pathogen or damage-associated molecular patterns, thereby activating downstream immune signaling pathways (Gao et al. 2025). Activation of these receptors initiates both local and systemic defense responses, such as cell death that is hypersensitive to salicylic acid and the development of systemic acquired resistance (SAR) (Mishra et al. 2024). Together, these responses form a coordinated defense system that prevents pathogens spread locally while preparing uninfected tissues for enhanced immunity. In parallel with biotic challenges, plants are continuously exposed to dynamic abiotic stresses such as drought, salinity, extreme temperatures, and heavy metal toxicity. These environmental stresses disrupt key physiological and biochemical processes, which lead to chlorosis, impaired photosynthesis, reduced reproductive success, and substantial low yield (Das et al. 2022; Agho et al. 2025). To mitigate these effects, plants employ a wide range of regulatory and metabolic strategies to restore cellular homeostasis and sustain growth under adverse conditions.

Phytohormones play a central role in coordinating plant adaptive responses to environmental stress. Compounds such as melatonin, γ-aminobutyric acid (GABA), jasmonic acid, salicylic acid, brassinosteroids, and strigolactones enhance stress tolerance by improving seed germination, plant growth, photosynthesis, root architecture, and antioxidant capacity, while simultaneously lowering ROS accumulation, malonaldehyde levels, and electrolyte leakage (Huang and Jin 2025). Among these, GABA and other non-protein amino acids are particularly effective in mitigating oxidative stress and maintaining metabolic homeostasis during drought, salinity, and temperature changes, especially in plants from the *Fabaceae* family (Hayat et al. 2012; Wang et al. 2025).

Adapting to a water deficit environment involves a combination of morphological and biochemical changes (Seleiman et al. 2021). Morphological traits such as deeper root systems, reduced leaf areas, and precise stomatal regulation contribute to improved water use efficiency. At cellular levels osmotic adjustment is achieved through the accumulation of compatible solutes, including proline, glycine betaine, and soluble sugars which help to maintain cell turgor under dehydration. Abscisic acid (ABA) plays a crucial role in promoting regulation of stomatal closure and increasing the activity of genes that respond to drought (Bharath et al. 2021). Salt stress creates additional ionic and osmatic problems for plants. To maintain cytosolic ion balance, plants compartmentalize excess Na⁺ into vacuoles and use specialized transport proteins like HKT, SOS1, and NHX to regulate ion homeostasis (Yuan et al. 2016). Halophytic species further improve salt tolerance by actively excreting ions through specialized salt glands allowing them to thrive in high-salinity environments (Yuan et al. 2016).

**Fig. 2:**
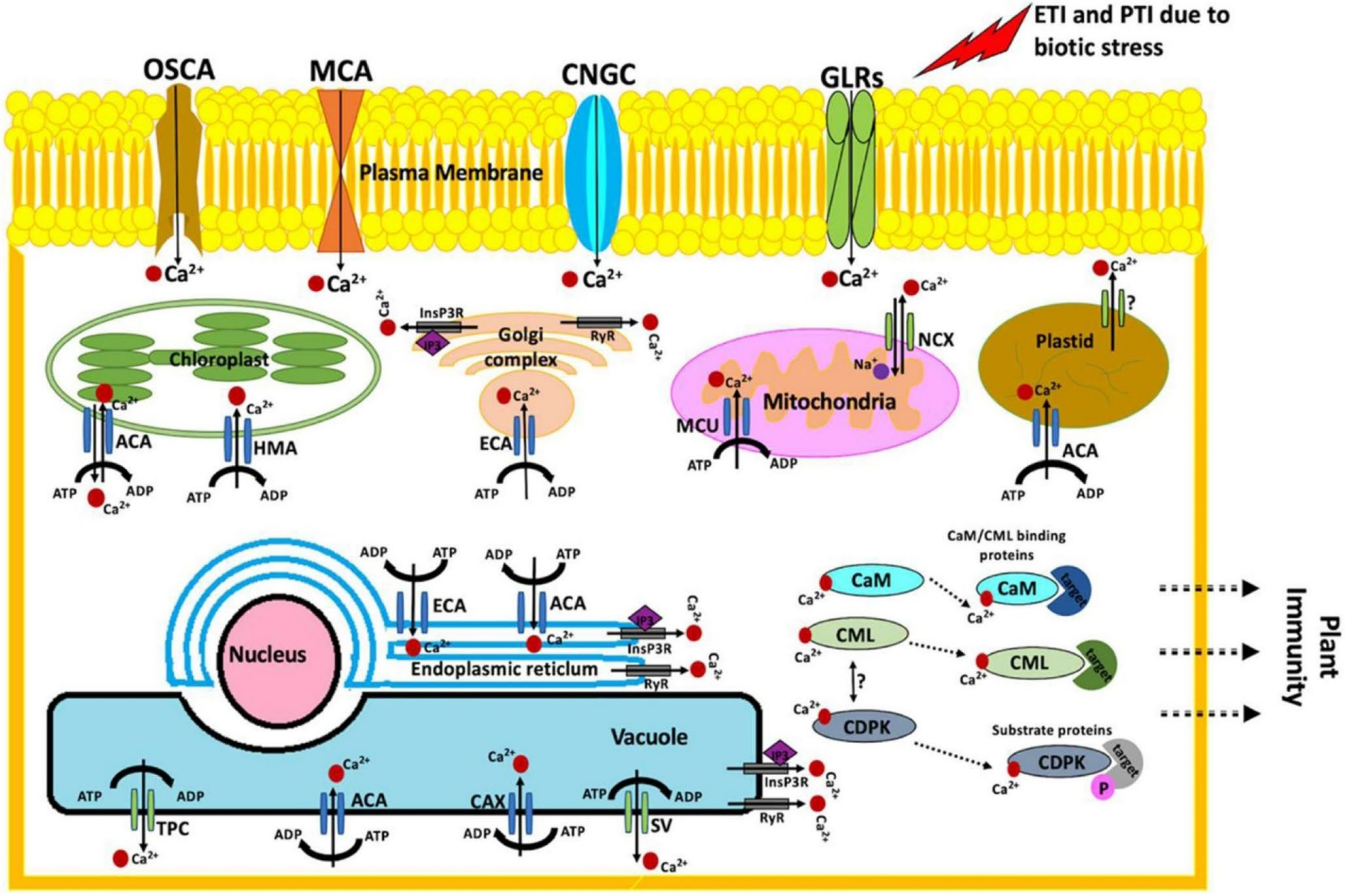
Diagrammatic illustration of Ca²⁺-mediated signaling pathways involved in the perception of biotic stress and the regulation of plant immune responses. Upon biotic stress recognition, cytosolic Ca²⁺ concentrations rise above basal levels (> 200 nM), activating Ca²⁺-dependent protein kinases (CDPKs), calmodulin (CaM), and calmodulin-like proteins (CMLs). Ca²⁺ influx, transport, and homeostasis are regulated by multiple channels and transporters, including cyclic nucleotide-gated channels (CNGCs), glutamate receptor-like channels (GLRs), stretch-activated Ca²⁺ channels (OSCAs), MID1-complementing activity channels (MCAs), two-pore channels (TPCs), mitochondrial Ca²⁺ uniporter (MCU), Ca²⁺ exchangers (CAX and NCX), P1-ATPases (e.g., HMA1), autoinhibited Ca²⁺-ATPases (ACAs), ER-type Ca²⁺-ATPases (ECAs), and vacuolar slow-activating channels (SV), along with secondary messengers such as inositol-1,4,5-trisphosphate (IP₃) and cyclic ADP-ribose (cADPR) acting via InsP₃R- and ryanodine receptor-like channels. These coordinated Ca²⁺ fluxes integrate energy and phosphate signaling (ATP, ADP, P) to fine-tune downstream immune responses, illustrating the central role of Ca²⁺ dynamics in linking biotic stress perception to plant defense activation. Reproduced with permission from Iqbal et al. (2021). Copyright © 2021 Frontiers (Open Access)

Extreme temperatures trigger plants to activate specific protective responses. Under cold conditions plants accumulate antifreeze proteins and activate cold-responsive (COR) genes, that help stabilize cellular components during freezing. In contrast, plants exposed to high temperatures depend on heat shock proteins (HSPs) to protect cellular proteins from denaturation and aggregation (Mas-ud et al. 2025). Heavy metal exposure presents an additional challenge, however, plants can limit toxicity by binding metals through chelation, depositing excess metal ions into vacuoles and producing antioxidant enzymes to counter oxidative damage (Mansoor et al. 2023). Overall, these responses indicate the ability of plants to sense environmental stress and activate coordinated defense mechanisms that maintain cellular function and support survival under adverse environmental conditions (Fig. 2).

### Signaling and molecular regulation

Calcium (Ca²⁺) serves as a pivotal second messenger in plant immunity, coordinating swift and highly specific signaling events in both effector-triggered immunity (ETI) and pattern-triggered immunity (PTI) (Vijayan 2025). When a pathogen is detected, a rapid influx of Ca²⁺ is triggered through multiple plasma membrane channels, such as OSCA, MCA, CNGC, GLRs (Fig. 2). These Ca²⁺ signatures are further shaped by intracellular stores, like chloroplasts (ACA, HMA), mitochondria (MCU, NCX), and plastids (ACA). In addition, the Golgi apparatus, endoplasmic reticulum, and vacuole act as dynamic reservoirs that regulate Ca²⁺ release and sequestration, thereby regulating cytosolic Ca²⁺ levels (Carvalho et al. 2020).

Elevated levels of Ca²⁺ in cytosol are sensed by Ca²⁺-binding proteins, including calmodulin (CaM) and CaM-like protein (CML), which decode these signals and activate downstream responses. A key outcome of this process is activation of Ca²⁺-dependent protein kinases (CPDKs), which connects Ca²⁺ signaling to changes in gene expression metabolism and immune responses. These kinases link Ca²⁺ signaling to gene expression, metabolism and the activation of immune response, ultimately coordinating the defense strategies that make up PTI and ETI (Shi et al. 2018).

### Antioxidant defense and stress signaling

Plants depend on coordinated antioxidant systems to prevent the excessive accumulation of ROS generated during environmental stress. Key antioxidant enzymes such as superoxide dismutase (SOD), catalase (CAT), and various peroxidases, convert harmful ROS intermediates into less toxic molecules. Through this process plants protect cellular structure and maintain redox balance (Forman and Zhang 2021). In addition, these biochemical defenses work together with stress-responsive genes that control ion homeostasis, osmolyte production, and other ROS-scavenging pathways ensuring metabolic stability under adverse conditions (Waheed et al. 2024). This molecular mechanism, phytohormones like ABA, JA and ET serve as central regulators that link environmental signals with downstream transcriptional programs, regulating plant responses to drought, salinity, and heat stress (Khan 2025).

### Adaptive and genetic mechanisms

**Fig. 3:**
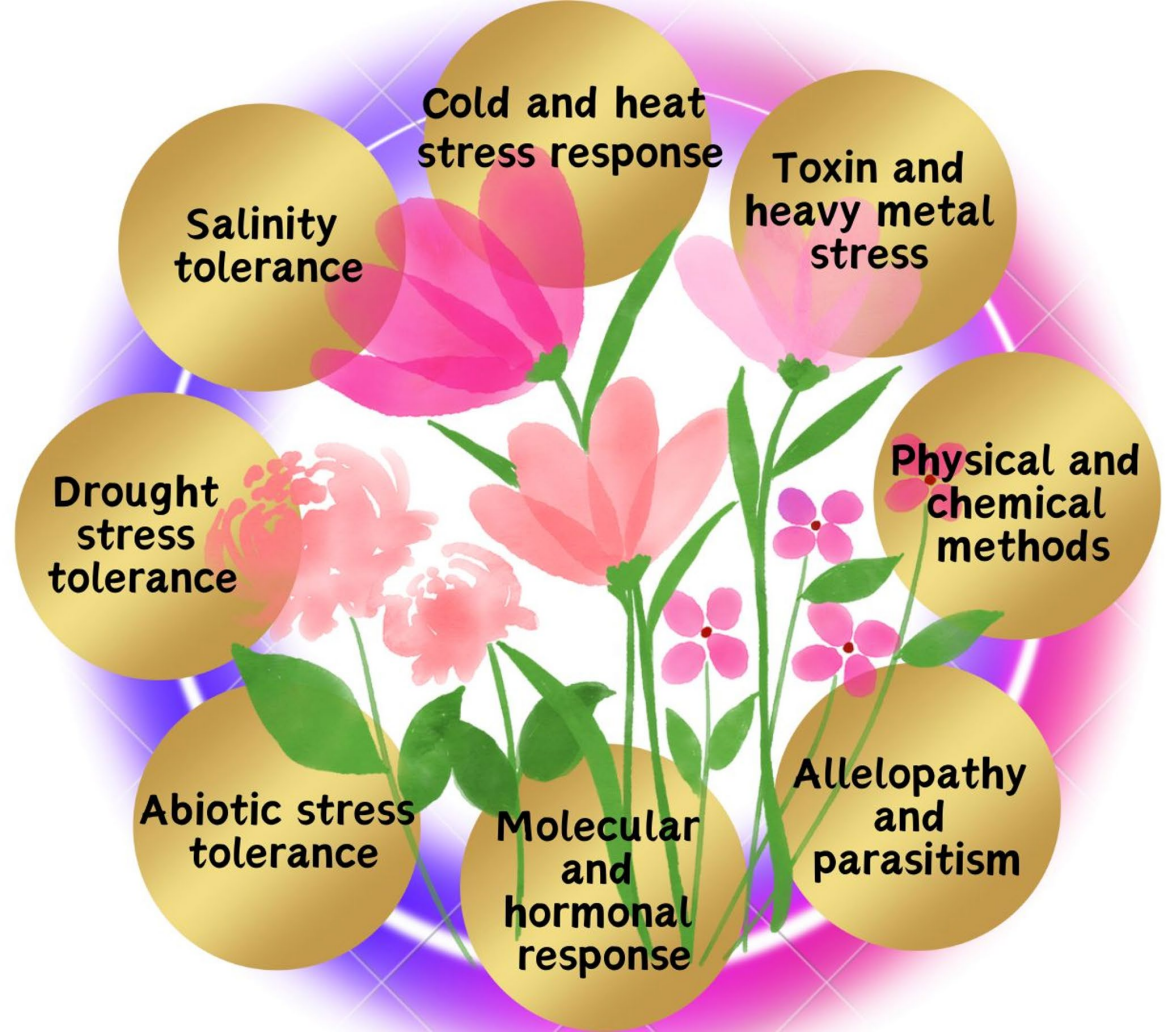
Diagrammatic representation of major abiotic stress factors, including drought, salinity, temperature extremes, and metal toxicity, that negatively affect plant growth and development. Plants respond to these stresses through coordinated molecular signaling networks, hormonal regulation, and physiological adaptations that enhance tolerance. The illustration highlights integrated defense strategies involving molecular and hormonal responses, physical and chemical protection mechanisms, and interactions such as allelopathy and parasitism, collectively emphasizing how multifaceted tolerance mechanisms enable plant survival under adverse environmental conditions

HSPs and antifreeze proteins are examples of adaptations to extreme temperature(Hu et al. 2022). Phytochelation, vacuolar sequestration, and increased antioxidant activity are all part of heavy metal tolerance (Shivappa et al. 2025). Improvement in genomics and transcriptomics make it possible to find genes that are related to stress(Adhimoolam et al. 2025) (Fig. 3). Plants have developed diverse strategies to protect themselves from pathogens, herbivores, and other environmental stresses. These defenses can be structural, chemical, molecular, or systemic and they play an important role in plant survival. The following table summarizes major types of plant defense mechanisms, providing their components, examples and specific functions (Table 1).

**Table 1:** Overview of plant defense mechanisms, summarising recognition strategies, signaling pathways, and downstream responses, along with their key benefits, inherent limitations, and unresolved knowledge gaps in achieving durable resistance against diverse biotic stresses

Type	Mechanism/component	Examples	Function	References
Physical defenses	Waxy cuticle, trichomes, thorns, lignified cell walls	Bark, lignin, spines	Prevent pathogen entry and herbivory	Watts et al. (2023)
	Bark and hard shells	Acacia thorns, nutshells	Provide a barrier and physical protection	Freeman (2008)
Chemical defenses	Phytoalexins, phenolics, terpenoids, alkaloids	Camalexin, flavonoids, tannins	Antimicrobial, antioxidant, insecticidal roles	Mithöfer and Boland (2012)
	Alkaloids & secondary metabolites	Quinine, caffeine, cyanogenic glycosides	Toxic and repellent to herbivores	Divekar et al. (2022)
	Saponins, glucosinolates	Mustard oil, saponins	Deterring pests and pathogens	Chhajed et al. (2020)
Molecular defenses	PR proteins, ROS-scavenging enzymes	Peroxidase, catalase, chitinase	Neutralize oxidative stress, degrade pathogen wall	Shobade et al. (2025)
	Cell wall strengthening	Callose deposition, lignification	Prevent pathogen penetration	Tiwari et al. (2025)
Signaling molecules	Hormones and messengers	SA (Salicylic Acid), JA, ET, H₂O₂, NO	Regulate defense gene expression	Sultana et al. (2024)
Indirect defenses	Attraction of natural enemies	Release of volatiles, extra floral nectar	Reduce herbivore numbers via predator attraction	Grof-Tisza et al. (2025)
Systemic responses	SAR and ISR	Induced by SA, rhizobacteria	Provide long-term, broad-spectrum protection	Basak et al. (2025)

### Signaling crosstalk and hierarchical defense responses

Brassinosteroids (BRs) and gibberellins (GAs) are the key phytohormones that regulate plant growth and development. In absence of GA, DELLA proteins accumulate in the nucleus, repressing growth-related transcription factors and restraining growth (De Bruyne et al. 2014). When GA is present, it binds to GID1, forming GA-GID1 complex that promotes DELLA ubiquitination and subsequent degradation. This releases growth-promoting transcription factors, enabling processes that are involved in stem elongation, germination, and flowering. Further, DELLA protein promotes plant defense. While they enhance resistance to biotrophs, their accumulation can reduce tolerance to necrotrophs. BR signaling operates through complementary mechanisms. In the absence of BRs, the kinase BIN2 remains active and suppresses the transcription factor BZR1. When BR is present, BIN2 is inactivated, allowing BZR1 to enter the nucleus and activate genes involved in cell elongation, vascular development and overall growth. Overall BZR1 can promote growth, but can also interact with immune regulators, enabling context-dependent defense response. DELLA proteins and BZR1 work together to balance growth and defense. Under high GA levels, DELLA degradation allows BZR1 to activate genes and promote development. When GA levels are low, stable DELLAs interact with BZR1 shifting the plant’s priority from growth toward defense. These changes ensure plant survival under unfavorable conditions.

Plant defense responses are further coordinated by SA, JA, and ET, which form a complex signaling network. SA-mediated signaling primarily regulates resistance to biotrophic pathogens, through the activation of systematically acquired resistance (SAR). Whereas JA and ET pathways are more closely linked to defenses against necrotrophic pathogens and herbivores insects (Li et al. 2019). A very complex signaling network controls plant defense with SA, JA, and ET as the main regulators.

**Fig. 4:**
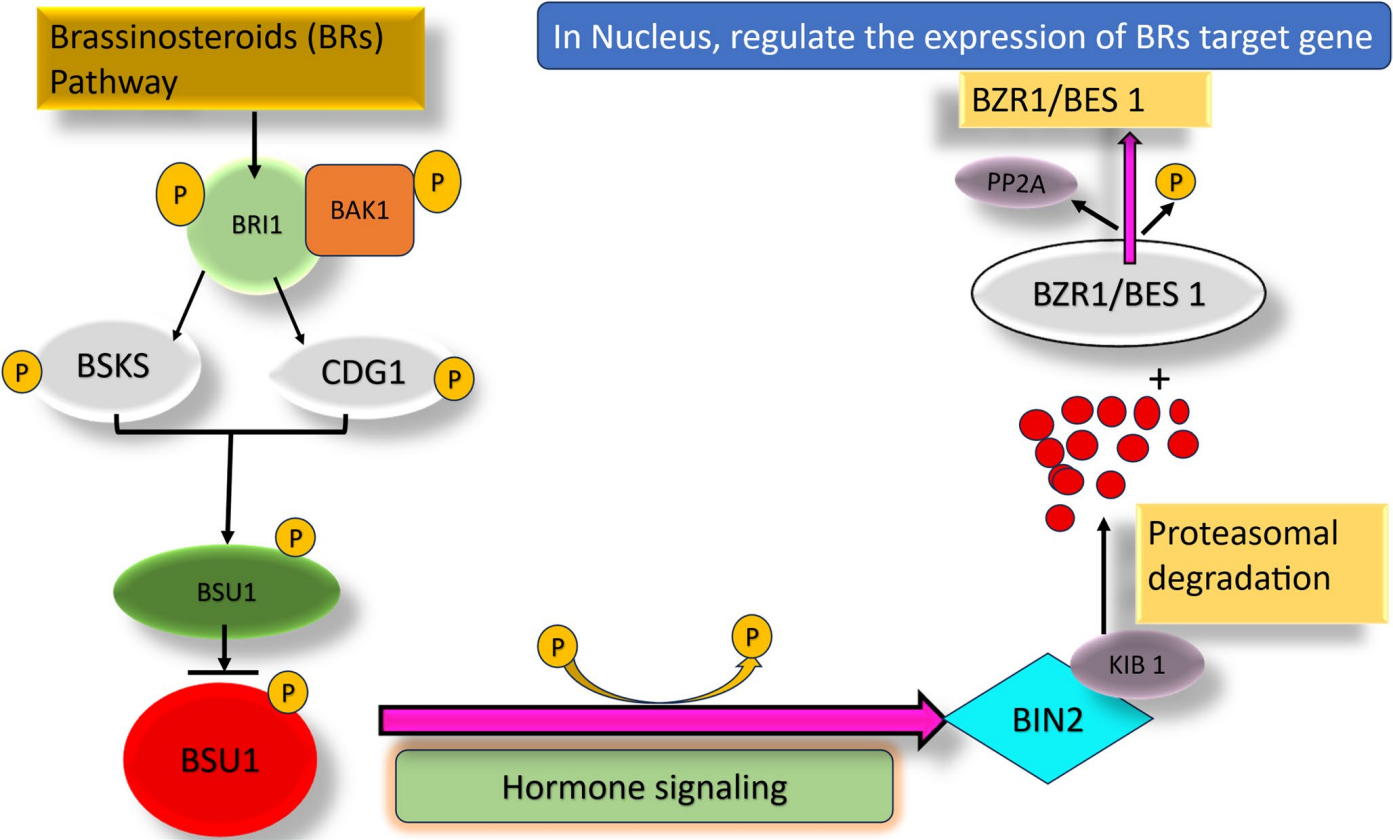
Schematic illustration of brassinosteroid (BR) signaling and its role in regulating plant growth and defense responses. Perception of BRs by the receptor BRASSINOSTEROID INSENSITIVE 1 (BRI1) and its co-receptor BAK1 initiates a phosphorylation cascade that inactivates the kinase BIN2, leading to activation of transcription factors BZR1 and BES1. These transcriptional regulators modulate BR-responsive gene expression, linking hormone perception to growth regulation while maintaining coordination with defense pathways

**Fig. 5:**
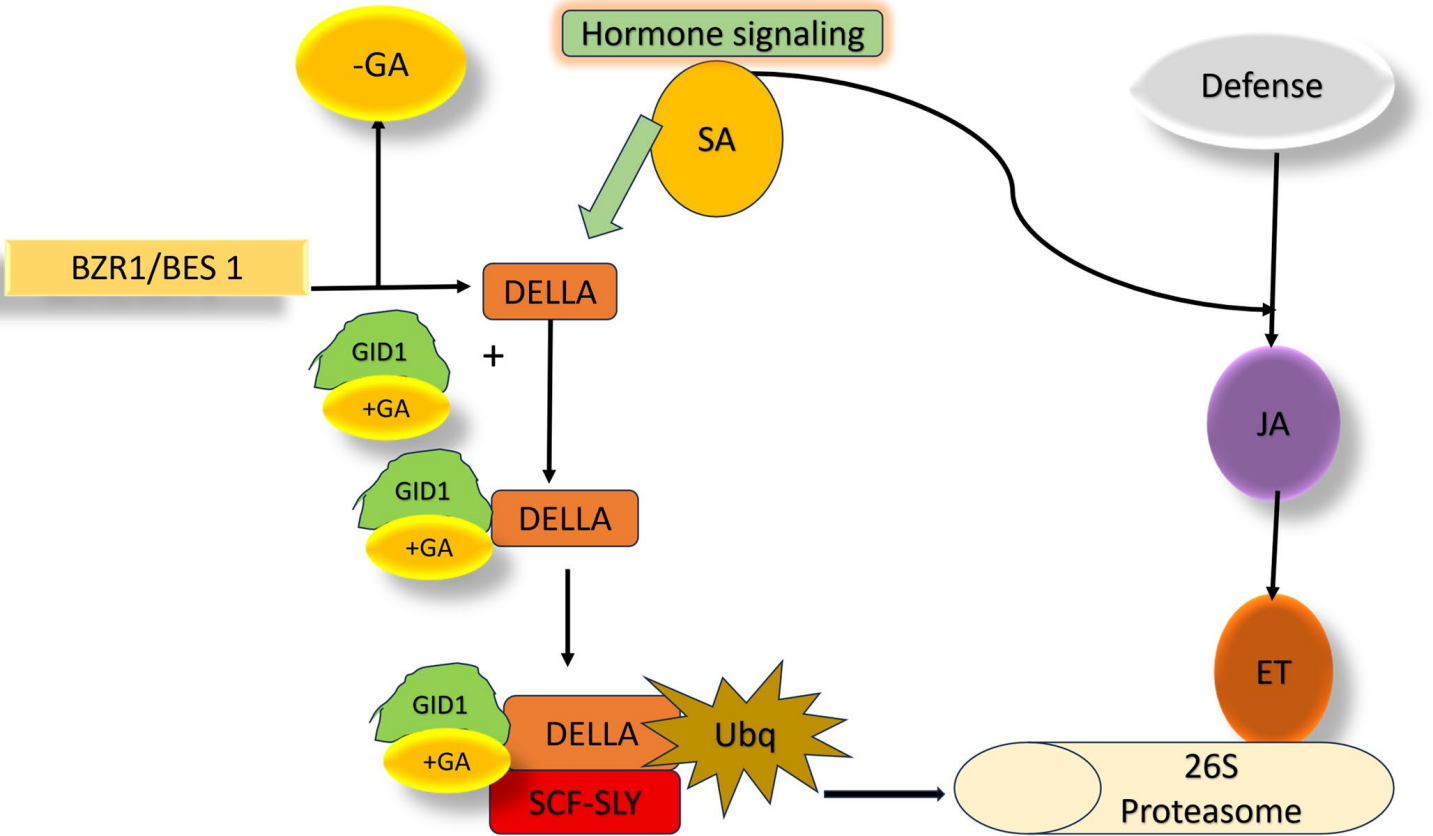
Overview of gibberellin (GA) signaling and its crosstalk with defense-related hormonal pathways. Binding of GA to its receptor GID1 promotes the degradation of DELLA proteins through the SCF-SLY complex and the 26 S proteasome, thereby enhancing growth responses. Under low GA conditions, DELLA accumulation suppresses growth while promoting defense through interactions with salicylic acid (SA) signaling, with additional modulation by jasmonic acid (JA) and ethylene (ET) pathways, highlighting the dynamic balance between growth and immunity

These pathways engage in extensive crosstalk that allows plants to fine-tune their immune responses according to the nature of the threat (Ding et al. 2022). SA-mediated signaling primary regulates resistance to biotrophic through the activation of systemic acquired resistance (SAR), whereas JA and ET pathways are more closely associated with defense against necrotrophs and herbivorous insects. Crosstalk between SA and JA/ET usually establishes an antagonistic relationship, which helps plants to conserve energy and prioritize appropriate defenses. For instance, strong SA signaling can suppress JA dependent responses when biotrophic pathogens dominate. Conversely, JA and ET often act synergistically to enhance resistance against tissue-damaging necrotrophs and herbivores (Li et al. 2019; Mur et al. 2006) (Figs. 4 and 5).

## Plant cell wall dynamics and immune response to pathogen infections

The plant cell wall is a critical barrier against pathogen invasion and frequently gets stronger through processes like thickening and lignification during infection (Kaur et al. 2022). Upon pathogen attack, pattern recognition receptors (PRRs) detect pathogen-associated molecular patterns (PAMPs) which triggered PAMP-triggered immunity (PTI). This early immune response promotes accumulation of structural reinforcements and the production of antimicrobial compounds that restrict pathogen entry and spread (Yu et al. 2017).

Quantitative studies have demonstrated that genes involved in lignin biosynthesis, such as phenylalanine ammonia-lyase (PAL), cinnamate-4-hydroxylase (C4H), and cinnamyl alcohol dehydrogenase (CAD) are strongly upregulated during pathogen infection, leading to enhanced lignification of the cell wall. For example, red leaf blotch (RLB) diseases of almonds (*Prunus dulcis*), caused by *Polystigma amygdalinum*, exhibits differential susceptibility among cultivars. In the tolerant cultivar (Mardía) early activation of *CAD* and *DFN1* genes resulted in increased deposition of lignin at the sites of fungal colonization, effectively restricting pathogen spread through both physical and chemical barrier. In contrast, the susceptible cultivar (Tarraco) showed delayed activation of genes such as *HQT* and *LDOX*, which are involved in chlorogenic acid and anthocyanin biosynthesis. However, the limited lignin accumulation in this cultivar fails to halt pathogen progression, indicating the importance of timely lignification as a key resistance mechanism against RLB in almond (Zúñiga et al. 2019).

ROS production is an integral part of this defense. ROS directly damages pathogen cellular components and also function as signaling molecules to boost immune response (Jena et al. 2023). In parallel, plants use enzyme inactivation mechanisms to prevent pathogen-mediated degradation of proteins and cell walls, while secondary metabolites such as phytoalexins further suppress pathogen growth. The nucleus contributes to defense by facilitating DNA-proteins crosslinking, which enhances cellular structural stability during pathogen attack (Chen et al. 2022). Vacuole serve as storage compartments for toxic compounds and defense-related enzyme, whereas mitochondria and chloroplast supply energy and generate signaling molecules essential for immune activation. In addition, SAR signals are transmitted to neighboring tissues, preparing them for potential future infection (Bobik and Burch-Smith 2015).

**Fig. 6:**
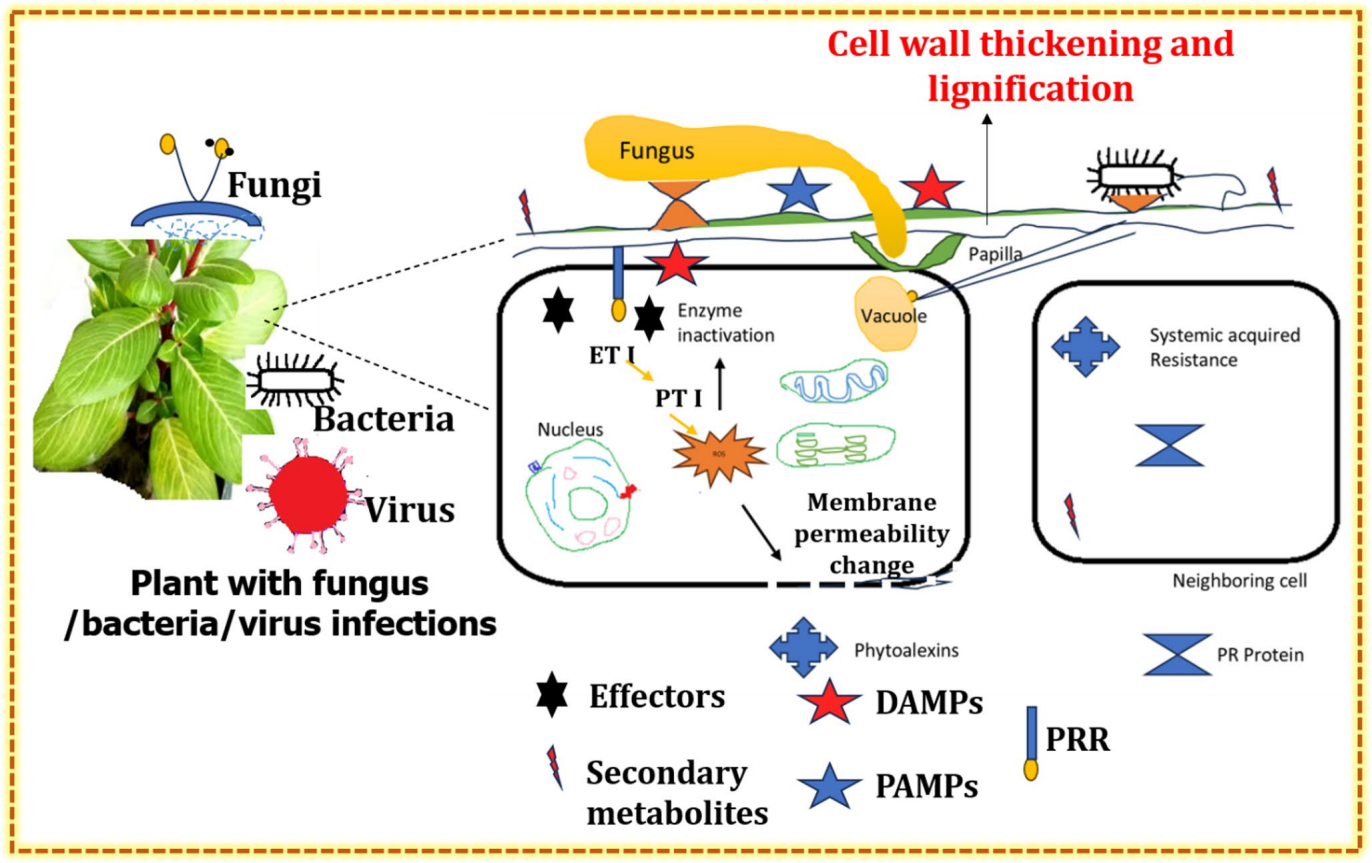
Schematic depiction of multi-layered plant immune responses activated upon pathogen attack by fungi, bacteria, or viruses. Recognition of pathogen-associated molecular patterns (PAMPs) initiates PAMP-triggered immunity (PTI), leading to defense responses such as cell wall reinforcement, lignification, enzyme inhibition, membrane permeability changes, and reactive oxygen species (ROS) generation. The subsequent development of systemic acquired resistance (SAR), characterized by the accumulation of pathogenesis-related (PR) proteins and phytoalexins, restricts pathogen spread and contributes to long-term plant immunity

Effector-triggered immunity (ETI) provides an additional layer of protection, wherein the recognition of pathogen-derived effectors proteins triggers strong localized defense response, including programmed cell death, to mitigate the infection. Together the structural, metabolic, and signaling processes demonstrate the highly coordinated nature of plant immune response, strengthening the central role of cell wall in pathogen protection (Ghosh et al. 2020) (Fig. 6).

Lignin biosynthesis is among the most complex and tightly regulated pathways in plant secondary metabolism. Lignin is a high-molecular weight phenolic polymer that strengthens the cell wall, confers rigidity and hydrophobicity and protects plants from both biotic and abiotic stresses. Beyond its structural function, lignin plays a critical role in tissue differentiation, organ development and stress resistance (Liu et al. 2018).

Lignin biosynthesis is regulated by an intricate signaling network involving phytohormones, such as ABA, JA, SA, ET, and auxin, as well as ROS, mitogen-activated protein kinases (MAPKs), and transcription factors from the NAC, MYB, and WRKY families. These interactive networks carefully control the activation of lignin biosynthetic enzymes such as phenylalanine ammonia-lyase (PAL), cinnamyl alcohol dehydrogenase (CAD), caffeic acid O-methyltransferase (COMT), and peroxidases (PODs) (Liu et al. 2018; Zhao et al. 2021).

Lignin biosynthesis is not isolated metabolic pathway. Instead, functions as a central hub where hormonal, redox, and transcriptional signals integrate to regulate plant growth, defense, and environmental adaptation. This dynamic interaction ensures that lignification is continuously adjusted in response to developmental cues and stress conditions, allowing plants to optimize both survival and performance (Table 2) (Sulis and Wang 2020).

**Table 2:** Role of nanotechnology in enhancing plant resilience, presenting specific plant examples and associated agricultural applications, while comparing the benefits, practical limitations, and knowledge gaps related to efficacy, environmental safety, and long-term field performance

Nos.	Approach	Nanoscience component	Biotechnology application	Examples	References
1	Nano-drug delivery	Polymeric nanoparticles, liposomes	Targeted drug/gene delivery	Liposomal doxorubicin, siRNA carriers	Patle and Dongre (2025)
2	Biosensing	Quantum dots, gold nanoparticles	Advanced biosensors/diagnostics	Quantum dot immunoassay	Derichsweiler et al. (2025)
3	Tissue engineering	Nanofiber scaffolds, nano-hydroxyapatite	Tissue regeneration	Nanofiber vascular grafts	Huang et al. (2025)
4	Agricultural enhancement	Silver/silica nanoparticles	Smart fertilizers/pesticides	Nano-silica for crop protection	Murugan et al. (2025)
5	Vaccine technology	Nanoemulsions, virus-like particles	Nanovaccine development	mRNA vaccines in lipid nanoparticles	Cheng et al. (2025)
6	Food safety & detection	Nano-biosensors	Detect foodborne pathogens/toxins	Gold nanoparticle biosensor	Rovina and Wen Xia (2025)
7	Environmental biotechnology	Magnetic nanoparticles	Bioremediation, pollutant removal	Magnetic nano-adsorbents	Bharti et al. (2025)
8	Personalized medicine	Surface-modified nanoparticles	Precision diagnostics/therapies	Targeted cancer nanomedicines	Tanvi et al. (2025)
9	Synthetic biology	DNA origami, nano-carriers	Gene circuits, artificial cells	DNA origami for cell design	Kumawat and Pathak (2025)
10	Smart implants	Nano-coatings, carbon nanotubes	Improved implant biocompatibility	Antibacterial nano-coated titanium implants	Kumawat and Pathak (2025)

### Basic plant physiological strategies

Plants depend on plant physiological strategies to survive, adapt, and sustain growth under varying environmental conditions. These strategies are designed to optimize photosynthesis, water use, and enhance stress tolerance, thereby maintaining overall plant productivity. For instance, photoprotection helps plants to minimize cellular damage caused by excessive light exposure. In arid environments, specialized photosynthetic pathways such as crassulacean acid metabolism (CAM) allow plants to open their stomata at night, reducing water loss while maintaining carbon fixation. Similarly, the C4 photosynthetic pathway increases carbon fixation efficiency by minimizing photorespiration, which enhances plant growth at higher light intensity and elevated temperatures. Together, these physiological adaptations contribute to improved plant resistance and productivity particularly under challenging environmental conditions (Khan et al. 2025).

Plants employ diverse carbon fixation strategies, including C3, C4, CAM, C4-CAM, and intermediates, indicating long-term adaptation to different ecological niches. C3 photosynthesis is the most common mode, in which carbon dioxide is directly fixed into a three-carbon compound (3-phosphoglycerate) by the enzyme Rubisco. However, this process becomes less efficient under high temperature and drought due to increased photorespiration (South et al. 2019). C4 photosynthesis evolved as an adaptation to hot and arid environment; it spatially segregates carbon fixation and the Calvin cycle by initially converting carbon dioxide into a four-carbon compound (oxaloacetate) in mesophyll cells. This compound is then transported to bundle sheath cells to enhance photosynthesis efficient (Burgess and Wang 2022).

CAM photosynthesis represents another adaptation to dry conditions and is commonly found in succulents, orchids, and cacti. In this pathway, stomata open at night to fix carbon dioxide into organic acids which is stored in vacuoles and later used for photosynthesis during the day when the stomata are closed to conserve water (Lüttge 2010). Some plant species exhibit C4-CAM flexibility, allowing them to switch between C4 and CAM pathways in response to environmental conditions thereby optimizing water use and growth (Gilman et al. 2022).

In addition to carbon fixation strategies, plants use photoprotective mechanisms to limit damage caused by excessive light and ROS. These include non-photochemical quenching (NPQ), the xanthophyll cycle and antioxidant defense systems. Excess excitation energy is dissipated as heat, pigments such as violaxanthin are converted to zeaxanthin and both enzymatic antioxidants are produced. Together these responses protect the photosynthetic machinery (especially PSII) from photoinhibition and oxidative damage (Zavafer et al. 2015).

## Integrating biotechnology and nanoscience into plant resilience

Plants depend on coordinated network of biotic and abiotic stress responses to adapt and thrive under adverse condition. These responses extensively involve the integration of phytohormonal regulation with emerging nano-biotechnological approaches, which together enhance stress resistance while supporting ecological balance.

### Advances in molecular biology and genetic engineering

Recent advances in molecular biology and gene-editing technologies have significantly expanded the potential for developing stress-resistant crops. In particular CRISPR/Cas systems, enable precise modification at genomic loci associated with drought salinity, temperature stress, and pathogen resistance. For instance, targeted modulation or overexpression of transcription regulators like *DREB1A* has been shown to enhance tolerance to water-deficit conditions in several transgenic crops.

Similarly, molecular breeding approaches, such as marker-assisted selection (MAS) identifies and propagates crops with better resistance traits (Devi et al. 2017). Together, gene-editing and molecular breeding technologies accelerate the development of climate-resistance crop varieties with improved productivity and nutritional value.

### Role of nanomaterials in stress tolerance

Nanomaterials have demonstrated significant potential in enhancing plant stress tolerance by improving nutrient absorption, agrochemical distribution and modulation of physiological processes. Metal and metal oxide nanoparticles particularly those based on zinc and titanium, have been reported to alleviate drought and salinity tolerance by improving photosynthesis and antioxidant enzyme activity (Zaman et al. 2025). Beyond these direct physiological effects, certain nanoparticles can alter the balance of phytochrome and improve the plant’s intrinsic defense systems, thereby improving overall stress adaptation.

### Antioxidant systems and ROS regulation

Reactive oxygen species (ROS) function as both damaging agents and signaling molecules in response to stress exposure. Plants reduce the harmful effects of ROS by using an antioxidant network that includes both enzymatic and non-enzymatic antioxidants. Enzymatic antioxidants include superoxide dismutase (SOD), catalase (CAT), and ascorbate peroxidase. Non-enzymatic antioxidants include flavonoids, carotenoids, and ascorbic acid (Rao et al. 2025). This mechanism maintains cellular redox homeostasis and prevents oxidative injury under adverse conditions. Emerging evidence suggests that some nanoparticles enhance antioxidant responses, reducing ROS accumulation and improving overall stress tolerance (Hasanuzzaman et al. 2020).

### Light-mediated and structural adaptations

Light perception and signaling mechanisms are crucial in modulating plant responses to environmental variations. Phytochromes manage important growth processes like germination, avoiding shade, and flowering. They also help plants to optimize light utilization (De Wit et al. 2016). Structural adaptations and extra layers of durability. For example, xxeromorphic leaves reduce water loss through transpiration in arid environments and the formation of aerenchyma improves oxygen diffusion in waterlogged soils (Mohanta et al. 2023). These morphological traits, along with light-mediated signaling, allow plants to adjust the balance between growth and defense.

### Nanotechnology-enabled gene delivery systems

**Fig. 7:**
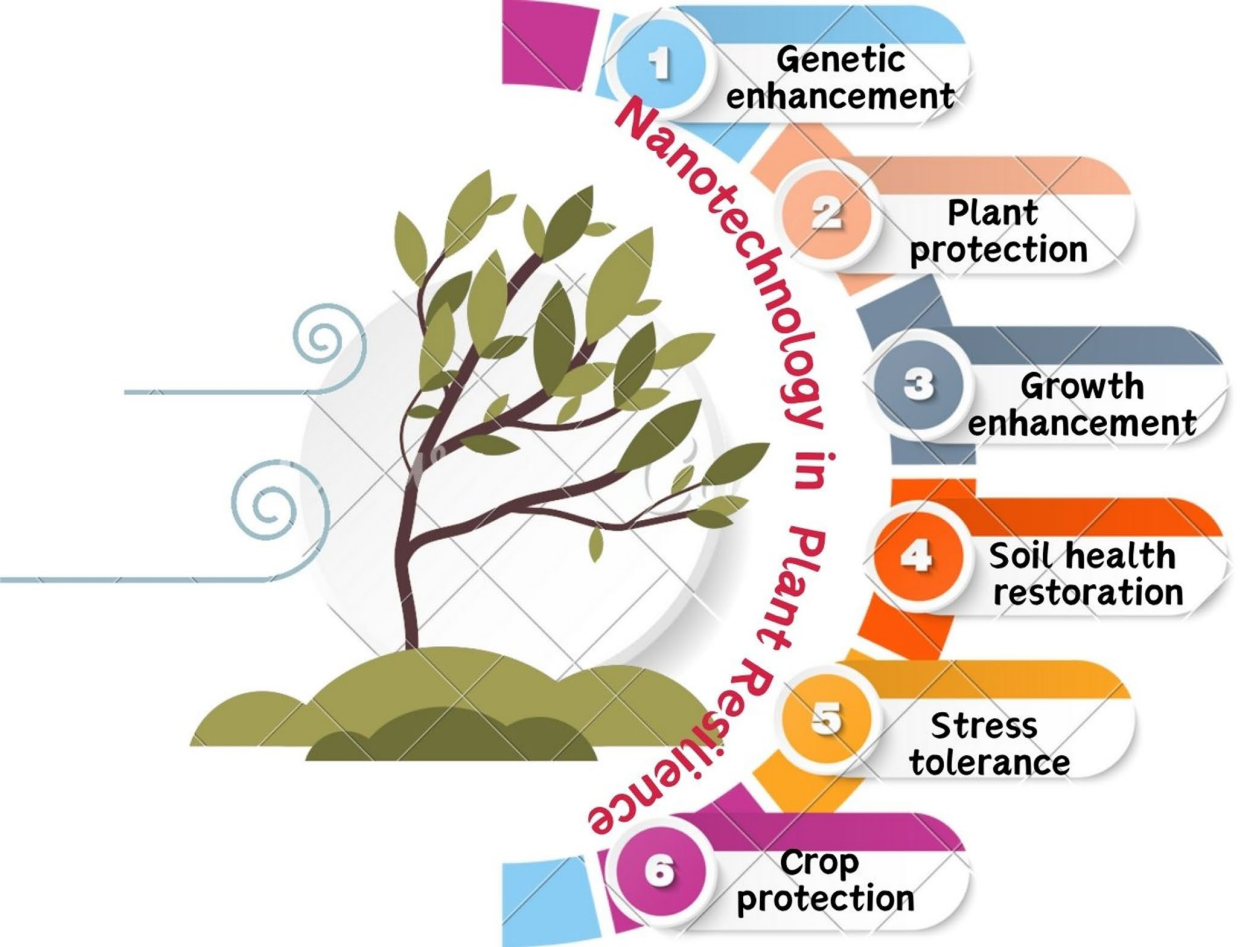
Diagrammatic overview of nanotechnology-based approaches for sustainable agriculture. Nanomaterials support genetic improvement, plant protection, growth promotion, stress tolerance, soil health restoration, and crop productivity through precise nutrient delivery, enhanced resistance mechanisms, and improved physiological performance. These integrated nano-enabled strategies collectively enhance plant resilience against both biotic and abiotic stresses while promoting sustainable agricultural practices

Nanotechnology is transforming plant genetic engineering by enabling precise, efficient and non-invasive delivery platforms. Carbon nanotubes, mesoporous silica nanoparticles and other nanocarriers can deliver DNA, RNA or CRISPR components directly into plant cells, avoiding traditional methods such as *Agrobacterium*-mediated transformation or gene guns. These systems that use nano-career make transformation more efficient and allow more species to be genetically modified. This makes it easier to use advanced molecular tools in crop improvement (Fig. 7).

Biotechnology and nanoscience are coming together to make major advancements in many disciplines, including agriculture, medicine and environmental engineering. Nanoscale materials-like metal and polymeric nanomaterials, quantum dots and nanofibers exhibited distinctive physicochemical properties that facilitate highly targeted multifunctional and efficient biotechnological interventions. Their incorporation into biological systems supports the advancement of next-generation diagnostics, therapeutics, biosensors, and intelligent agricultural technologies, accelerating expediting the movement toward personalized medicine, sustainable agriculture and environmentally conscious innovation (Table 3).

**Table 3:** Interdisciplinary applications of nanoscience and biotechnology, highlighting representative approaches, functional components, and application examples, with an emphasis on their advantages, constraints, and existing gaps that hinder large-scale agricultural translation

S. nos.	Application	Nanoparticle used	Mode of action	Plant (Botanical name)	Plant defense mechanism	References
1	Genetic enhancement	Carbon nanotubes	Gene delivery for stress resistance	Maize (*Zea mays*)	Basal immunity (PTI, ETI); systemic acquired and induced resistance activation	Khan et al. (2024)
2	Early blight control (fungal)	Silver NPs (AgNPs)	Induce host-resistance gene expression; inhibit *Alternaria solani*	Tomato (*Solanum lycopersicum*)	PR gene upregulation; enhanced defense enzyme activity	Kumari et al. (2017)
3	Bacterial disease protection (broad)	Silica NPs (SiO₂-NPs)	Induce SAR via salicylic acid pathway; PR1/PR5 up	Arabidopsis (*Arabidopsis thaliana*)	SAR activation via SA signaling; ROS-mediated defense	El-Shetehy et al. (2020)
4	Fusarium wilt suppression	Salicylic-acid–doped iron nano-clusters (Fe-SINCs)	Prime SA signaling; enhance antioxidative capacity; restrict fungal invasion	Watermelon (*Citrullus lanatus*)	SA-pathway activation; antioxidant enzyme enhancement	Noman et al. (2024)
5	Innate immunity boosting (general, demonstrated in tea leaves)	Chitosan NPs (CNPs)	Elevate defense enzymes (PO, PPO, PAL, β-1,3-glucanase); increase NO; upregulate defense genes	Tea (*Camellia sinensis*)	Induced resistance via NO signaling and PR activation	Chandra et al. (2015)
6	Bacterial spot control (*Xanthomonas* spp.)	Copper-based NPs (Cu/CuO)	Bactericidal activity; reduce disease severity in planta	Tomato (*Solanum lycopersicum*)	Reduced pathogen load; enhanced systemic protection	Varympopi et al. (2022)
7	Salinity-stress resilience + disease-related redox fortification	Zinc oxide NPs (ZnO-NPs)	Boost antioxidant enzymes (SOD, CAT, APX) under stress	Maize (*Zea mays*)	Antioxidant defense activation; ROS scavenging	Seleiman et al. (2023)
8	Spot blotch mitigation & growth promotion	Selenium NPs (SeNPs)	Activate enzymatic & non-enzymatic defenses; enhance resistance	Wheat (*Triticum aestivum*)	Defense enzyme activation; increased phenolic antioxidants	Shahbaz et al. (2023)
9	Bacterial wilt mitigation	Silica NPs (SNPs/SiO₂-NPs)	Activate defense responses; physiological & molecular changes vs. *Ralstonia solanacearum*	Tomato (*Solanum lycopersicum*)	Defense gene activation; improved immunity	Wang et al. (2022)
10	Rice blast control (“plant-vaccine” approach)	Carbon dots (CDs)	Prime resistance; reduce *Magnaporthe oryzae* (blast)	Rice (*Oryza sativa*)	Induced resistance and immune priming	Lei et al. (2025)
11	PR/defense activation by phyto-AgNPs	Phytofabricated AgNPs	Upregulate PR genes in roots & shoots; metabolite shifts	Tomato (*Solanum lycopersicum*)	PR gene induction; immune signaling modulation	Ashraf et al. (2025)
12	Early blight control in tomato (fungal)	Green-fabricated AgNPs (Q. incana extract)	Strong antifungal activity in vitro and on leaflets; >92% inhibition of *Alternaria solani*	Tomato (*Solanum lycopersicum*)	Pathogen inhibition; phenolic and enzyme-based defense	Khatoon et al. (2024)
13	Enhanced defense biochemistry in tomato	AgNPs–fluconazole conjugate nanoparticles	Synergistic increase in defense-related biochemical markers post-infection (e.g., phenols, SOD, PAL)	Tomato (*Solanum lycopersicum*)	Antioxidant and phenolic defense activation	Mirajkar et al. (2025)
14	Fusarium wilt control (tomato)	PP-AgNPs (phytofabricated)	Reduced disease severity; upregulated PR and defense genes; antifungal efficacy assessed in field	Tomato (*Solanum lycopersicum*)	Antioxidative and PR response activation	Ashraf et al. (2025)
15	Rice blast resistance (“plant-vaccine” style)	Carbon dots (CDs)	Enhanced systemic resistance via IAA and JA signaling, stabilized chloroplasts, boosted photosynthesis	Rice (*Oryza sativa*)	Systemic resistance; hormonal signaling and resilience	Lei et al. (2025)

### Genetic enhancement

Nanotechnology has changed approaches to genetic improvement in plants by making precise and efficient gene delivery systems. Nanomaterials such as carbon nanotubes and mesoporous silica are nanoparticles and used to deliver DNA and RNA directly into plant cells, bypassing traditional methods like *Agrobacterium* or gene guns. This method overcomes species-specific limitations and allows genetic modification across a wider range of crops. Nanoparticles, for instance can deliver CRISPR/Cas components, facilitating targeted genome editing to enhance traits such as drought resistance, pest resistance, and yield improvement. At nanoscale, these carriers protect genetic material from enzymatic degradation, improving delivery efficiency and stability. In addition to introducing beneficial traits, nanoparticle-mediated delivery systems are being explored for silencing deleterious genes or modulating stress-responsive pathways.

Collectively these advances open new avenues for developing resilient crop varieties capable of thriving in extreme climatic conditions while reducing dependence on chemical inputs. Future advancements are expected to integrate nanogenomics with precision agriculture to create sustainable farming solutions (Zaman et al. 2025).

### Plant protection

Nanotechnology plays an important role in protecting plants from both biotic and abiotic stress. Nano-formulated pesticides, herbicides, and fungicides enable controlled release of active ingredients, ensuring effective protection with minimal chemical use. For example, nanoencapsulation protects active compounds from rapid degradation prolonging their efficacy. Certain nanoparticles such as silver and zinc oxide contain antimicrobial properties, protecting plants from pathogens. In addition, nanosensors are being developed for early detection of pests and diseases, allowing timely intervention before severe damage. Nanocoatings applied to seeds can further improve germination, seedling can further improve germination, seedling vigor and tolerance to environmental stress. By minimizing pesticide leaching and runoff, nanotechnology supports more environmentally sustainable agricultural practices. Together, these innovations offer promising eco-friendly strategies for crop protection in modern farming systems (Alam et al. 2024).

### Growth enhancement using fertilizer

Nanofertilizers are engineered to deliver nutrients to plants more efficiently while minimizing losses and environmental waste. Unlike conventional fertilizers, nanofertilizers enable the slow and sustained release of nutrients, reducing the frequency of application. Nanoparticles such as hydroxyapatite and zeolites can adsorb and gradually release key nutrients such as nitrogen, phosphorus, and potassium in a controlled manner.

This controlled nutrient availability enhances uptake efficiency, supports root development and ultimately promotes better growth and higher yields. Nanoformulations can be used to deliver micronutrients such as zinc and iron to address widespread nutrient deficiencies in crops. By improving nutrient-use efficacy and reducing fertilizer usage, nanofertilizer support environmentally sustained farming practices while contributing to long-term food security (Saurabh et al. 2024).

### Soil health restoration

Nanotechnology offers promising solutions for restoring degraded soils and improving soil fertility. Nanoparticles can be used for remediation of contaminated soil by capturing or degrading heavy metals, hydrocarbons, and pesticides. For instance, iron oxide nanoparticles can adsorb lead and cadmium toxic metals, preventing their uptake by plants. Nanocatalysts can accelerate the breakdown of persistent organic pollutants, converting them into non-toxic forms. Beyond remediation, nanotechnology can also enhance soil health by supporting beneficial soil health by supporting beneficial soil microbiota. By creating more favorable conditions for microorganisms involved in nutrient cycling and soil aggregation, nanomaterials contribute to improved soil structure and fertility. Material such as nanoclays and biochar further enhance soil water retention and aeration, which support root growth and boost crop productivity. Collectively, these applications highlight the potential of nanotechnology to mitigate soil degradation and promote sustainable land management (Sharma and Kumar 2024).

### Stress tolerance with nanoparticles

Nanoparticles have emerged as effective tools for improving plant tolerance to abiotic stresses including drought, salinity, and extreme temperature. Metal and metal oxide nanoparticles such as zinc oxide and titanium dioxide help to enhance plant antioxidant system by improving ROS scavenging, thereby reducing oxidative damage. Silicon nanoparticles contribute to cell wall reinforcement and improve drought tolerance by limiting water loss. Under saline conditions, nanoparticles help to regulate ion uptake and maintain ionic balance, reducing salt-induced toxicity, they also play a role in reducing heat stress by stabilizing the photosynthetic machinery and protect enzymes from denaturation (Zaman et al. 2025).

### Crop protection using nanopesticides

Nanopesticides represent a major advancement in plant protection technologies, by enabling more precise targeting of pests and pathogens. Nanoformulations allows active ingredients to be released in controlled manner, reducing the frequency and quantity of pesticide applications required. For instance, polymeric nanoparticles protect pesticides from degradation by UV light and microbial activity, thereby extending their effectiveness. Metallic nanoparticles, such as silver and copper possess intrinsic antimicrobial properties, offering dual benefits of pest control and disease prevention. In addition, nanosensors integrated with nanopesticides systems enable real-time monitoring of pest population. These advancements reduce pesticide residues in crops and the environment, promoting safe and sustainable agricultural practices (Izuafa et al. 2025). The role of nanotechnology in enhancing plant resistance across species and stress conditions are summerized in Table 4.

**Table 4:** Integrated signaling networks regulating lignin biosynthesis in plants, outlining key molecular components, regulatory interactions, and functional outcomes, together with their benefits for stress tolerance, limitations in pathway manipulation, and gaps in Understanding cross-pathway coordination

Nos.	Mechanistic aspect	Functional connection	References
1	Phenylpropanoid pathway initiation	Lignin originates from the phenylpropanoid pathway, beginning with *PAL* and *C4H*. This entry point is regulated by hormonal signals such as ABA and JA during abiotic stress.	Liu et al. (2018)
2	Hormonal coordination hub	Multiple hormones-ABA, SA, JA, ET, auxin, and brassinosteroids-act synergistically or antagonistically to modulate lignin biosynthetic genes for defense or development.	Xie et al. (2018)
3	ABA-mediated lignification	Under drought or salt stress, ABA upregulates *PAL*, *4CL*, and *CAD*, enhancing lignin deposition for xylem stability and water retention.	Ma (2024)
4	JA–SA antagonism	JA induces lignin synthesis in wounding and insect defense, while SA modulates it during pathogen attack, creating a dynamic defense–growth balance.	Xie et al. (2018)
5	ROS as dual regulators	Reactive oxygen species act as signaling molecules and oxidative substrates for peroxidases and laccases in lignin polymerization.	Denness et al. (2011)
6	MAPK signaling integration	MAPKs (MPK3, MPK6) transduce abiotic and biotic stress signals, activating TFs like WRKY, MYB, and NAC that regulate lignin enzyme genes.	Yan et al. (2015)
7	NAC–MYB regulatory cascade	NAC and MYB transcription factors form a hierarchical network controlling *COMT*, *CCR*, and *CAD* genes-central to lignin biosynthesis.	Nakano et al. (2015)
8	Feedback between ROS and lignin	Lignin deposition reduces oxidative stress, while ROS generation promotes lignification-forming a positive feedback loop.	Yaqoob et al. (2022)
9	Omics evidence of signaling crosstalk	Transcriptomic and metabolomic profiling reveal concurrent activation of phenylpropanoid and MAPK pathways under stress, confirming multi-signal convergence.	Steffen et al. (2024)
10	Environmental triggers	Abiotic stresses (drought, UV-B, cold, metals) induce lignin biosynthesis via ROS-ABA-NAC–MYB signaling circuits to strengthen tissue tolerance.	Deng et al. (2024)

Nanotechnology has emerged as a powerful tool in modern agriculture, particularly for strengthening plant resilience to biotic and abiotic stresses. A wide range of nanoparticles and nanoformulations have been developed to enhance genetic traits, improve growth conditions, and protect plants from environmental challenges. For example, carbon nanotubes are used for delivering genetic material in crops like *Zea mays* (Maize) enabling improved stress tolerance (Zaman et al. 2025). In *Solanum lycopersicum* (Tomato), nano-silica protects plants from pest, while zinc oxide nanoparticles improve nutrient uptake and promote optimal growth in *Triticum aestivum* (wheat) (Grewal et al. 2024). Nanotechnology has also shown promise in improving soil-related stresses. Iron oxide and sulfur nanoparticles have been used to remediate contaminated soil and reduce heavy metal stress in *Brassica juncea* (Indian Mustard) (Praveen et al. 2018). Similarly, silica nanoparticles improve water retention and osmatic regulation in in *Oryza sativa* (rice), reducing drought stress (Rakesh et al. 2024). Titanium dioxide nanoparticles stabilize heat shock protein, in *Capsicum annuum* (Chili Pepper) (Alabdallah et al. 2024), while gold nanoparticles have been shown to induce cold-responsive proteins and improve cold stress resistance in *Arabidopsis thaliana* (Venzhik et al. 2024).

Additional applications highlight the broad utility of nanomaterials across crop species. Nano-zinc oxide increases chlorophyll content and supports photosynthesis in *Hordeum vulgare* (Barley) during adverse conditions (Azarin et al. 2023). Nano-chitosan formulations have demonstrated effective targeted pest control in *Gossypium hirsutum* (Cotton), reducing environmental impact while improving efficacy (Kariyanna and Sowjanya 2025). Silver nanoparticles exhibit strong antifungal and antibacterial activity enhancing disease resistance in *Allium cepa* (Onion) (Hegazy et al. 2025).

Beyond plant protection, nanotechnology contributes to soil restoration. Carbon-based nanoparticles improve microbial interactions and organic matter breakdown in *Lens culinaris* (Lentil), supporting sustainable soil management (Hegazy et al. 2025).Nanoparticles-mediated regulation of phytohormone has been shown to enhance growth and stress tolerance in crops such as *Cucumis sativus* (Cucumber) (Chandrashekar et al. 2023). Furthermore, nano-zinc formulation protects plants from UV stress in *Solanum tuberosum* (Potato) (Singh and Gill 2024), while water-efficient nano-polymers reduce water wastage in arid cultivation of *Cicer arietinum* (Chickpea) (Singh et al. 2025).

### Feasibility of nanotechnology in high- and low-income agricultural systems

The feasibility of integrating nanotechnology into agriculture varies widely between high-income and low-income farming countries. In high-income countries modern infrastructure, established regulatory frameworks, and strong financial investment facilitate rapid testing, validation, and commercialization of nano-enabled agricultural products. However, in low- income regions are often constrained by high production cost, weak extension services, lack of farmer awareness and socio-economic barriers. Despite these challenges, low-income agricultural systems stand to benefit substantially from affordable and sustainably synthesized nanotechnologies that enhance soil fertility and crop productivity. In South Africa studies have shown that nano fertilizers can improve nutrient-use efficiency by up to 30%, increase maize yields by 25%, and reduce nitrogen fertilizer requirements by 40%, while also improving soil health. These outcomes highlight the potential of nanotechnology to strengthen food security and promote sustainability across the continent (Khundi et al. 2025).

In Europe (EU) and Switzerland, clear guidelines govern on use of nanoparticles in agriculture. However, in many non-EU countries, such regulations remain unclear or absent, slowing wider adoption. However, nanoparticles that have been proven effective in biomedical application are increasingly being explored for agricultural use, where they can serve as direct source of micronutrients or as carrier for bioactive agrochemical, thereby enhancing crop growth, yield and stress resistance. These properties are strongly influenced by their physiological characteristics including metal composition, size, shape, surface chemistry, and coatings (Balusamy et al. 2023).

While high-income systems benefit from stronger support and funding for use of nanotechnology in agriculture, several low-income regions are beginning to explore locally adapted solutions. In India, nano-urea field trials have demonstrated reduced fertilizer cost and significantly improved crop yields, suggesting a cost-effective and scalable nano-solution tailored for low-resource settings.

Environmental processes such as aggregation, dissolution, surface transformation, and interactions with natural organic matter strongly influence the movement and bioavailability of nanomaterials in soil and aquatic ecosystems. Understanding the environmental fate of engineered nanomaterials is therefore essential for sustainable use. Nanoparticle transport and aggregation behavior are largely governed by size, surface charge, morphology, and surface functionalization.

Studies examining long-term and chronic exposure indicate that even at low concentrations of nanomaterials may exert adverse effects on biological systems. These effects include oxidative stress, membrane damage, genotoxic responses, alteration in soil microbial communities, and disruption of nutrient cycling processes. Evidence of trophic transfer and bioaccumulation has further raised concern about potential impacts on higher organisms and long-term stability. Despite growing research, there is limited understanding about how nanoparticles interact with co-existing pollutants, behave under real-world environmental conditions, and affect organisms across multiple generations. Consequently, comprehensive life-cycle assessments and long-term ecotoxicological studies are critical before the large-scale environmental deployment of nanotechnology-based solutions (Singh et al. 2025).

Although nanotechnology shows substantial potential in lab conditions, several technical and economic challenges limit its widespread application. Nanoparticle synthesis often requires high energy inputs, expensive precursors and stringent control to ensure batch consistency, while also generate secondary waste. Green or biogenic synthesis approaches offer environmentally friendly alternatives, but these methods frequently face limitations related to yield, process control and scalability. Conversely, traditional physicochemical synthesis methods frequently raise concerns about environmental burden and economic viability.

From a socioeconomic perspective, the successful implementation of nanotechnology depends not only on its technical performance but also on economic viability, infrastructure availability, regulatory clarity, and public acceptance. In developing and resource-limited regions, implementation is often constrained by high initial investment expenses and the absence of well-defined regulatory frameworks. However, emerging strategies that use locally available biomaterials, energy-efficient synthesis methods, and small-scale production systems offer promising pathways to improve affordability and regional accessibility. For nanotechnology to deliver meaningful benefits in environmental and agricultural solutions, its development needs to align with sustainability goals, supported by appropriate policy framework and guided by ethical, environmental and social factors (Malik et al. 2023).

## Comparing traditional vs. modern adaptive strategies

### Traditional adaptive strategies in plants

Plants rely on inherent defense mechanisms to defend themselves from biotic and abiotic stress. Central to these traditional strategies are phytohormone signaling pathways that coordinate stress perception and response. SA mediates resistance against biotrophic pathogens, while JA defends against necrotrophs and herbivores, and ET act as versatile regulator, integrating both biotic and abiotic stress responses and modulate crosstalk among hormonal pathways. Physical barriers form a first line of defense against invading pathogens and insects. Structures such as cuticle, trichomes, and lignified cell walls limit pathogen entry and reduce herbivore damage. These barriers are complemented by biochemical defenses, particularly the production of secondary metabolites. Alkaloids often function as toxins, phenolic compounds act as antioxidants and antimicrobials and terpenoids contribute to broad spectrum antimicrobial activity. Together, these metabolites strengthen plant resistance and reduce susceptibility to stress.

Plant exhibits a form of stress memory, which is generally short-term but can occasionally induce epigenetic modifications. Such changes allow plants or their progeny to respond more effectively to recurring stress, however, this capacity remains limited in comparison with engineered adaptations. Secondary metabolites play a central role in these adaptive responses, with their composition and abundance varying according to species, genotype, developmental stage, and environmental conditions. Increasing research over recent decades has focused on understanding how abiotic stresses, influence secondary metabolism in both in vivo and in vitro systems. Advances in molecular biology have clarified the signaling pathways regulating secondary metabolite biosynthesis, enabling targeted metabolic engineering of key intermediates (Isah and Isah 2019).

Salinity stress affects plants physiology, biochemistry, morphology, and metabolic pathways, largely through the induction of oxidative stress and ROS signaling. Molecular profiling studies have demonstrated that salinity significantly alters plant secondary metabolite composition in both crop and medicinal species. Additionally, salt stress frequently overlaps with drought responses, as high salt concentrations reduce root water uptake and induce osmotic stress, leading to solute accumulation and metabolic reprogramming (Parihar et al. 2015). Stress-inducible secondary metabolites such as terpenoids, flavanols, and flavones play important roles in plant-pathogen interactions and immunity and are increasingly explored as eco-friendly alternatives to synthetic pesticides.

These stress responses are tightly regulated by transcription factors (TFs) that regulate stress-responsive gene expression and secondary metabolite synthesis. Recent progress in transcriptional and post-transcriptional regulation highlights potential for enhancing secondary metabolite production molecular breeding and genetic modification. Despite substantial progress, the precise mechanisms underlying secondary metabolite-pathogen interactions remain incompletely understood and need further investigation (Anjali et al. 2023). In response to biotic stress imposed by pathogens and herbivores, plants use integrated defense systems involving structural barriers, biochemical deterrents, and hormone-mediated signaling. The zigzag model of plant-pathogen interaction shows how PTI and ETI work together to provide layered defense.

Traditional breeding and biotechnology have suggested this mechanism to develop resistant crops, such as Bt cotton and virus-resistant papaya, which reduced the use of chemical pesticides. More recently, approaches include CRISPR/Cas9, RNAi, and marker-assisted breeding enabled precise genetic modification that complement conventional breeding and enhance crop resistance, supporting sustainable agricultural practices (Umar et al. 2025).

Plants also employ physiological and symbiotic changes to withstand drought stress. Drought avoidance mechanism involves stomatal closure and leaf rolling to limit water loss and reduce transcriptional activity to conserve resources. Osmotic adjustment involves the accumulation of compatible solutes including proline and glycine betaine, which help to maintain turgor pressure, stabilize cellular structures, and support hydration under low soil moisture conditions. In addition, mycorrhizal symbiosis enhances water and nutrient uptake by extending root surface area and improving solute transport. Collectively, thereby adaptive responses enable plants to survive and maintain productivity under water-limited conditions (Sun et al. 2024).

### **Modern adaptive strategies in plants**

Modern approaches to enhancing plant stress tolerance integrate advanced molecular and biotechnological tools aimed at improving crop resistance and sustainability. Among these, CRISPR/Cas-mediated gene editing has emerged as a transformative technology, enabling precise modification of stress-responsive genes associated with drought, salinity, and temperature tolerance. Importantly, these edits can be introduced without incorporating foreign DNA, which makes it widely acceptable and regulatory feasibility. In parallel, transgenic overexpression of genes encoding antioxidant enzymes, stress proteins, and resistance genes directly enhance defense by increasing protective molecules and improving survival under oxidative and environmental stresses.

Synthetic biology further expands these capabilities by enabling the design of novel genetic circuits by building effect defense pathways that extend beyond natural mechanisms. Such approaches allow the construction of finely tuned and robust stress-response networks. In addition, epigenome editing offers a complementary strategy by modifying gene expression through targeted epigenetic marks without altering the DNA sequence. This approach prepares plants for stress and even passes on “stress memory” to future generations. Together, these modern approaches provide innovative routes for developing stress-resistance crops that support sustainable agriculture and climate adaptation (Kumar et al. 2023).

Cotton is a crop of major economic importance since it provides fiber, oil, and protein. However, climate change and rising global demand increases pressure on cotton. While conventional breeding has contributed to improved yield and quality, it is often limited by long breeding cycles, dependency on natural variation and extensive backcrossing. Genome editing technics such as CRISPR/Cas systems offer powerful alternatives due to their simplicity, precision and flexibility compared with earlier tools such as zinc-finger nucleases and TALENs. In cotton, CRISPR/Cas has successfully applied to enhance resistance to biotic and abiotic stresses, improve fiber quality, alter plant architecture and flowering, and enable gene stacking with minimal segregation issues. The transgene-free methods further increase their acceptability for practical use (Fiaz et al. 2021).

**Fig. 8:**
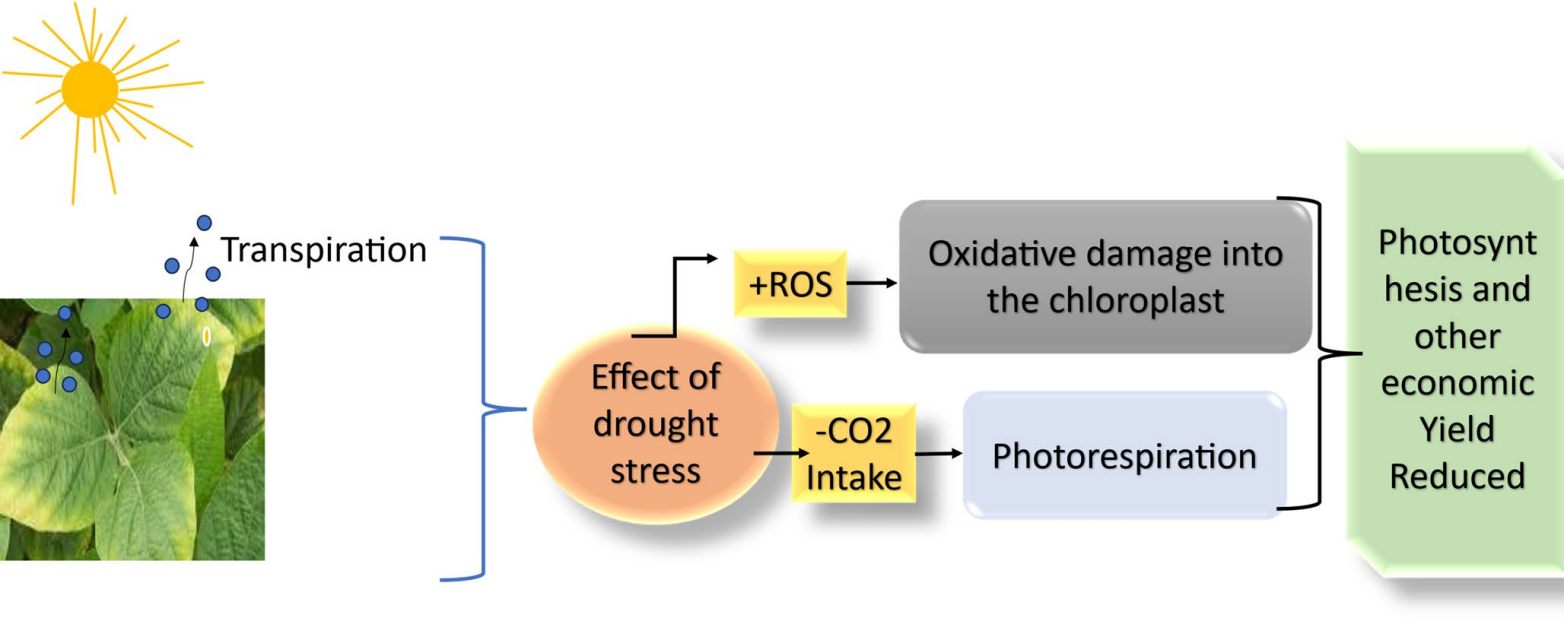
Illustration of drought-induced metabolic and physiological alterations in plants. Water deficit leads to excessive accumulation of reactive oxygen species (ROS), resulting in oxidative damage, increased photorespiration, reduced CO₂ assimilation, and impaired photosynthetic efficiency. Drought stress further disrupts ATP synthesis and induces structural and functional damage to cellular components, ultimately contributing to reduced crop productivity

**Fig. 9:**
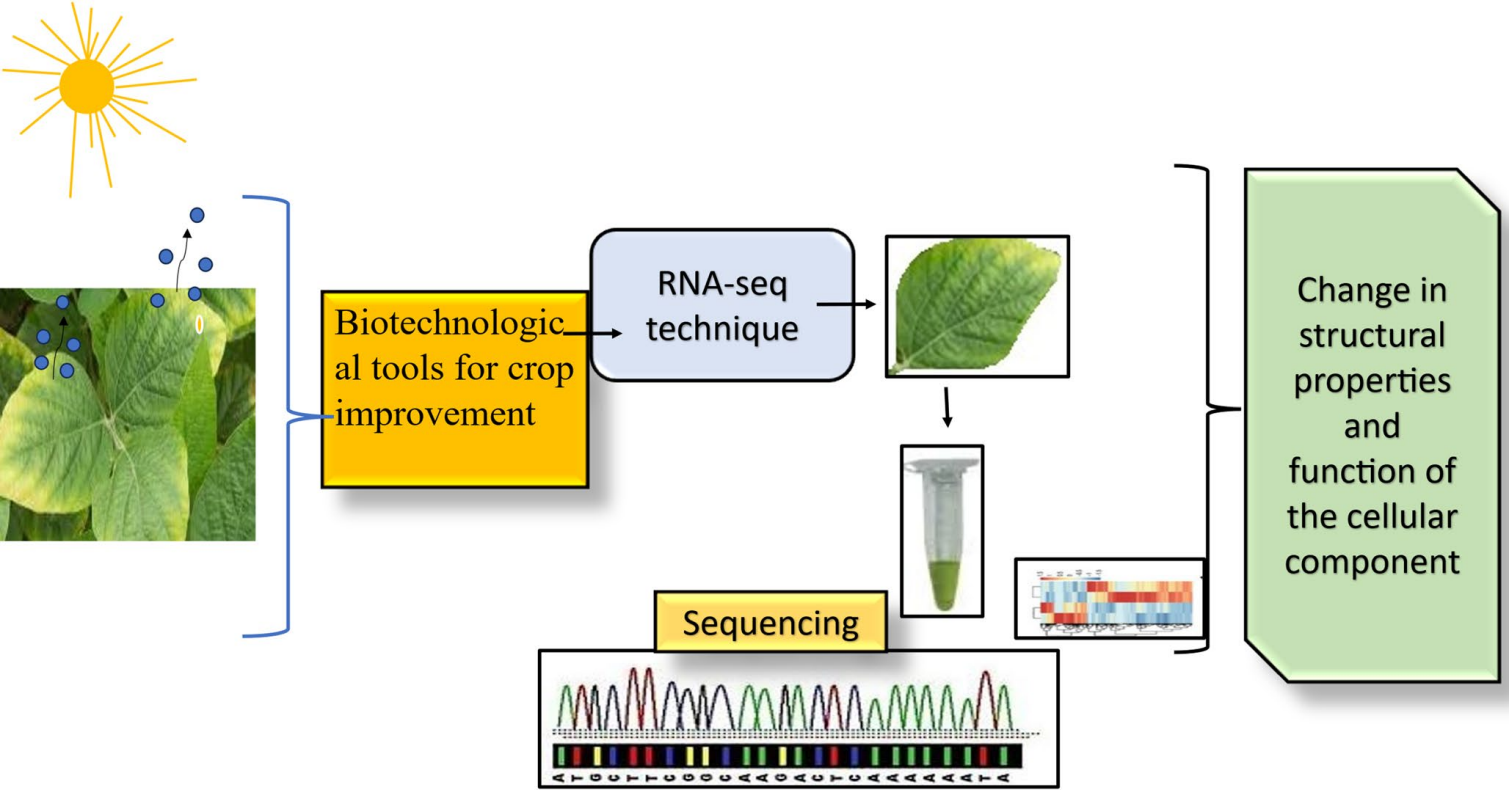
Schematic representation of modern biotechnological tools used to elucidate plant stress responses. Advanced approaches such as RNA sequencing (RNA-seq) and genomic analyses enable the identification of stress-responsive genes, regulatory networks, and molecular targets, providing critical insights for developing crop varieties with enhanced drought tolerance and stress resilience

Nanotechnology has become an integral component of modern agricultural strategies, working with genetic engineering and molecular biology to improve resource management and stress tolerance. Nanofertilizers deliver nutrients in controlled and sustained release forms, increasing uptake efficiency while reducing nutrient losses and environmental pollution. Similarly nano hydrogels act as water reservoirs in soil retaining large volume of water and releasing it gradually to plants. Advances in genetic engineering have additionally allowed modification of root architecture, producing deeper and highly branched root systems that enhance water and nutrient absorption. Targeted regulation of aquaporins, specialized membrane channel proteins, further improves internal water transport, contribute to better hydration and stress tolerance. Collectively, these strategies provide sustainable solutions for efficient resource use in modern crop production (Yadav et al. 2023a, b) (Figs. 8 and 9).

### Traditional and modern strategies: socioeconomic feasibility of nanotechnology

For decades, traditional agricultural practices such as selective breeding, organic fertilizers, and conventional pest management have played a central role in improving crop productivity and sustainability (Karavolias et al. 2021). While these methods have contributed significantly to sustainable farming, their effectiveness is very limited by environmental stress, resource depletion, and rapid climate change. In contrast modern strategies, particularly those that use nanotechnology, on the other hand, offer accuracy, responsiveness and long-term benefits by enabling targeted input delivery and real-time crop management (Ijaz et al. 2023).

Nanofertilizers, nanopesticides, and nanosensors facilitate controlled nutrient release, efficient pest management, and real-time monitoring of plant health. These technologies reduce input wastage, minimize environmental contamination, and improve efficiency in resource use. However, agriculture remains a critical driver of economic growth in both developed and developing countries and modern agricultural practices have disrupted ecological balance by affecting nutrient cycles, soil health, and carbon sequestration. In this context, organic farming continues to represent an environmentally sustainable alternative by avoiding synthetic fertilizers and pesticides, enhancing soil fertility, improving food quality, and promoting biodiversity. These principles align closely with global frameworks for sustainable agriculture (Gamage et al. 2023).

Evidence suggests that integration of biotechnological and nanotechnological methods can deliver substantial economic and environmental benefits. Farmers adopting such advance practices have reported yield increases of up to 33% and net income gains of approximately 60%, leading to reduced input costs and lower environmental impacts. Higher benefit-cost ratios improved technical efficiency and more equitable income distribution among adopters indicate both economic viability and social benefits. Scenario analysis further suggests that wider adoption could significantly increase revenue and environmental savings, reinforcing the role of modern technologies in achieving sustainable and efficient crop production systems, such as chickpea farming (Das et al. 2025).

Driven by global climate change and rising food demand, agriculture is undergoing a paradigm shift toward sustainability and technological innovation. Although traditional farming methods are important, they often fall short in addressing issues like declining soil fertility, pest resistance, and resource inefficiency. Integrating nanotechnology with innovation management offers a transformative approach to overcoming these challenges by enhancing input efficiency, improving crop resistance, and restoring environmental balance. Identified innovation clusters provide practical guidance for improving productivity, efficiency and environmental sustainability (Dabare et al. 2025).

The socioeconomic feasibility of nanotechnology depends on its ability to deliver measurable societal and economic benefits while ensuring equitable access, affordability, and sustainability. Conventional feasibility assessments mainly emphasized technical performance and cost-effectiveness (Rodriguez et al. 2023). In contrast, modern methods encompass multidimensional assessments, integrating social inclusivity, ethical governance, environmental sustainability, and long-term innovation management. This shift reflects alignment with the broader objectives of sustainable development goals (SDGs) (Kanashiro Uehara 2024).

Nanotechnology is a rapidly evolving, multidisciplinary field that combines diverse areas spanning agriculture, pharmaceuticals, and engineering. Despite its substantial technological and economic promise, concerns persist regarding potential environmental and health risks, particularly under conditions of large-scale deployment. Public awareness of these risks remains limited, while industrial and pharmaceuticals sectors continue to invest heavily in nanotechnological applications, sometimes without fully addressing long-term safety implications. Consequently, balancing innovation with responsible governance, risk assessment, and transparent regulation remains essential to ensure that the benefits of nanotechnology over its potential risks (Yadav et al. 2013) (Table 5**).**

**Table 5:** Comparison of conventional and sustainable models for nanotechnology assessment in agriculture, evaluating their benefits, limitations, and outstanding knowledge gaps related to environmental impact, regulatory frameworks, and long-term sustainability

Nos.	Parameter	Traditional strategy	Modern strategy	References
1	Evaluation focus	Economic profitability and industrial growth	Sustainability, social equity, and environmental safety	Pandey and Jain (2020)
2	Decision-making model	Top-down (policy or corporate driven)	Participatory governance with public–private–community partnership	Malakar et al. (2022)
3	Economic assessment	Focused on ROI and market share	Integrates life-cycle cost, sustainability, and circular economy	Mpongwana and Rathilal (2022)
4	Social inclusion	Limited to industrial stakeholders	Involves farmers, local entrepreneurs, and consumers	Yadav et al. (2023a, b)
5	Innovation ecosystem	Closed innovation (patent monopoly)	Open innovation, PPPs, and tech incubation networks	Gu et al. (2021)
6	Environmental considerations	Often neglected or secondary	Green synthesis, eco-toxicological evaluation, biodegradability	Nizam et al. (2021)
7	Regulatory framework	Fragmented or absent	ISO, OECD, and REACH-based standardized nano-safety policies	Allan et al. (2021)
8	Assessment tools	Manual economic surveys and feasibility studies	AI, big data, and bibliometric tools (PRISMA, VOSviewer) for analysis	Pokrajac et al. (2021)
9	Global technology transfer	Concentrated in developed economies	South–South and North–South collaborations for equitable access	Kumar et al. (2024)
10	Alignment with SDGs	Weak or indirect linkage	Strong alignment with SDGs 2, 7, 9, 12, and 13	Elzein (2024)

## Conclusion, future directions and challenges

Plants have evolved an extremely advanced system to sense, respond to, and adapt to a wide spectrum of environmental stresses. These built in defense mechanisms including multilayer signaling cascades, cell wall remodeling, and immune responses, that enable them to recognize and respond to diverse stresses. These inherent strategies operate across structural, physiological, biochemical, and genetic levels, and have long supported crop productivity and food security. However, the increasing intensity and complexity of climate-driven stresses increasingly exceeds the limits of natural plant adaptation demands advanced intervention strategies.

Recent progress in nanotechnology, CRISPR-based genome editing, and molecular breeding have opened new opportunities for strengthening plant stress tolerance with greater precision. These methods enable targeted modulation of stress-responsive pathways, improved nutrient and water use, and enhanced resistance to both biotic and abiotic stresses. By integrating traditional plant defense mechanism with cutting-edge molecular and nano based technologies, it is now possible to improve crop productivity, sustainability and food security. However, the feasibility and impact of these innovations vary across socio-economic contexts. While technologically advanced agricultural systems may readily adopt nano-enabled genome-editing strategies, resource-limited regions require solutions that are affordable, scalable, and socially acceptable.

Despite significant progress, several critical challenges and research opportunities remain. Greater emphasis is required on integrating traditional defense mechanisms with emerging nanotechnological and genetic approaches. In this regards, engineered nanomaterials (ENMs) have gained attention for their potential to modulate plant immunity by enhancing antioxidant and defense-related pathways, and influencing plant-associated microorganisms. Despite these advances, significant challenges remain regarding their mode of action, long-term environmental impacts, biodegradability, and targeted delivery efficiency.

A deeper understanding of how nanomaterials and genome-editing tools interact with plant signaling networks, redox homeostasis and immune regulation is essential. This assists in optimizing their efficacy while minimizing unintended physiological and ecological effects. Issues of scalability and socioeconomic feasibility are equally important, particularly for large-scale deployment in low- and middle-income agricultural systems. In parallel, understanding on environmental fate, bioaccumulation, and non-target effects of nanomaterials and gene-edited crops remain limited and require systematic investigation.

Finally, improving crop performance under multiple, concurrent stresses such as drought, heat, salinity, and pathogen pressure, remains a major challenge. Addressing this complexity will require integrative breeding strategies supported by precision agriculture. Successful translation of these technologies will depend on harmonized regulatory frameworks, transparent ethical evaluation, and increased public engagement. The integration of genomics, phenomics, and data-driven modeling approaches can further enhance stress prediction and decision-making, ultimately support sustainable, climate-resilient agriculture while protecting environmental and societal interests.
